# Sourdough authentication: quantitative PCR to detect the lactic acid bacterial microbiota in breads

**DOI:** 10.1038/s41598-017-00549-2

**Published:** 2017-04-03

**Authors:** Erica Pontonio, Raffaella Di Cagno, Jennifer Mahony, Alessia Lanera, Maria De Angelis, Douwe van Sinderen, Marco Gobbetti

**Affiliations:** 1 Department of Soil, Plant and Food Sciences, University of Bari A. Moro, Bari, Italy; 2 Faculty of Science and Technology, Free University of Bolzano-Bozen, Bolzano, Italy; 3 School of Microbiology, Cork, Ireland; 4 APC Microbiome Institute, University College Cork, Cork, Ireland

## Abstract

No national legislation anywhere in the world regulates and protects traditional/typical sourdough breads. Sourdough fermentation is firmly associated with a century-old tradition, and with sensory and nutritional quality of breads. A well-defined cell density of lactic acid bacteria has to be reached at the end of fermentation, and be indirectly detectable in baked breads. A Quantitative PCR (qPCR) method was developed to discriminate between breads made with and without sourdoughs. Universal primers targeting an approximately 178-bp fragment of the 16S rRNA-encoding gene of lactic acid bacteria were designed, covering the known diversity of sourdough lactic acid bacteria and excluding commonly encountered flour bacterial contaminants. A total of 191 breads either made with traditional type I and dried sourdough and baker’s yeast, or by a chemical leavening method were shown to be accurately discriminated by means of qPCR. Discriminating values of gene copy number were only weakly correlated with pH values, and with lactate and acetate concentration, thus questioning the validity of these latter indirect indices. The use of sourdough has to be guaranteed to meet both bakery and consumer expectations, and to fulfil legal requirements; our work presents a reliable authentication method providing a suitable tool to satisfy such requirements.

## Introduction

Food authentication relies on the verification of stated specifications of a given food. This may involve numerous aspects, including the validation of processing protocols. Food authentication and food fraud detection are the two opposing sides of the same coin and serve to protect the consumer^1^. Leavened baked goods and, in particular, breads are staple foods that are consumed globally, with local variations in recipe, processing, packaging and presentation. On average, the annual European consumption of bread is approximately 58 kg *per capita*, varying from 120 kg in Turkey to 32 kg in the UK^2^. About 30 to 50% of the breads manufactured in European countries require the use of sourdough^3^. In Italy, ca. 200 different types of traditional/typical sourdough breads are manufactured, especially by small or medium-size specialized bakeries^4^. Sourdough represents one of the most ancient uses of a natural starter, where contaminating (i.e., not deliberately added) lactic acid bacteria and yeasts grow and co-exist. In recent years, an astonishing number of scientific reports have in one way or another reported on various sourdough aspects: some 1,200 published items were retrieved from the main literature databases in January 2017. A consensus on the capacity of sourdough to positively influence the sensory, nutritional, texture, and shelf-life characteristics of baked goods is now well established^5, 6^. Increased knowledge on the application of this natural starter has catalyzed its technological transfer and promoted the growing global manufacture of sourdough breads, reflecting old traditions and satisfying consumer demands. Although the term sourdough is legally protected in several European countries (e.g., Germany, France, Austria, Denmark, Sweden and Norway)^7–10^, to date, the authentication of sourdough breads solely relies on the self-declaration by manufacturers as stated on food labels, the determination of indirect parameters (e.g., the pH value, or concentration of lactic and acetic acids) or the superior sensory and rheology quality subjectively perceived by consumers. Surprisingly, no currently applicable European regulation stipulates what lactic acid bacterial cell density has to be reached at the end of fermentation, and to be indirectly detected in baked breads where non-spore-forming cells are killed. This remains a crucial feature to clearly distinguish sourdough from other bread varieties. Although naturally co-existing with yeasts, lactic acid bacteria, in particular hetero-fermentative species, are not only the main distinguishing feature when compared to other leavening agents (e.g., chemical leavening and baker’s yeast), but are also primarily responsible for the superior quality of sourdough breads^5, 6^. The complexity and stability of the sourdough microbiota depend on a number of determinants, which include the flour microbiota, other ingredients (e.g. honey, apple and yogurt) and house microbiota^11^. The presence of key sourdough lactic acid bacteria as endophytes of wheat plants^12^ and as inhabitants of the animal/human intestinal tract^13, 14^ also appears to impact on the sourdough microbiota. Because of these non-controllable sources of contamination, the variety of traditional/typical protocols, and the exogenous and endogenous technology parameters for bread making^15, 16^, the lactic acid bacterial composition of sourdough microbiota is very diverse and only moderately stable over time. Despite this diversity, a consensus opinion was reached nearly twenty years ago^17^, stipulating that the lactic acid bacterial viable count has to reach at least 7.0 Log cfu/g at the end of sourdough fermentation. Furthermore, the industrial use of dried or pasteurized sourdough has been introduced as leavening agents in the late 1970^18^. Therefore, a method to assess lactic acid bacterial cell density subsequent to their elimination by the baking process is needed to authenticate sourdough bread. Such a method would take preference over the less reliable and, in several cases, non-feasible analysis of doughs before baking.

Authentication is one of the technological pillars on which food industries are reliant. Analytical techniques for food authentication are diverse, and depend on the type of process and product. PCR-based techniques, in particular quantitative PCR (qPCR), are the basis of some of the most significant advances in food diagnostics^19^. Examples of PCR-based applications are the authentication^20–22^ and detection of incorrect description of and frauds^23, 24^ involving meat products, and the detection of foodborne and beneficial microbes in foods^25, 26^. Regarding bakery products, PCR-based techniques have been used to monitor (i) wheat sequences of DNA extracted during milling and baking^27^, (ii) sourdough microbiota during fermentation^28^, and (iii) lactic acid bacteria in dried sourdoughs^29^.

To the best of our knowledge, qPCR has not previously been employed for the detection of sourdough lactic acid bacteria in breads, where baking temperatures exceed 100 °C, thus causing DNA denaturation. The current study describes a qPCR method for the reliable detection and quantification of the lactic acid bacterial community in breads, thus facilitating the reliable discrimination between breads made with or without sourdoughs.

## Results

### Biochemical characterization of breads

A total of 191 breads made under pilot plant conditions (laboratory) or collected from bakeries were characterized based on their pH value and total titratable acidity (TTA), as well as the concentration of lactic and acetic acid (Supplementary Table S1). Laboratory sourdough breads (86 samples) showed values of pH and TTA that ranged from 3.93 ± 0.01 (ALA5) to 6.10 ± 0.01 (V*d*1), and 2.2 ± 0.1 (ALA*d*1) to 13.4 ± 0.2 (ALA5) ml of NaOH (0.1 N), respectively. The concentration of lactic and acetic acid was in the range 1.8 ± 0.4 (V*d*1) to 28.8 ± 0.2 (MTA7) mmol/kg and 1.1 ± 0.1 (V*d*1) to 11.7 ± 0.6 (V3) mmol/kg, respectively. These differences depended on the percentage of sourdough used and the duration of fermentation, which, in turn, was shown to affect final lactic acid bacterial cell numbers and associated metabolic end products. Laboratory breads made with baker’s yeast (12 samples) as the only leavening agent, had values of pH and TTA that ranged from 5.99 ± 0.02 (BY4) to 6.25 ± 0.02 (BY9), and from 0.6 ± 0.1 (BY8) to 1.7 ± 0.1 (BY4) ml of NaOH (0.1 N), respectively. Nine out of 12 baker’s yeast breads did not contain detectable lactic and acetic acid, while the remaining three baker’s yeast breads had levels of these organic acids at a concentration that was slightly below 1 mmol/kg.

Sourdough breads (57 samples) collected from bakeries were determined to have pH and TTA values that ranged from 3.94 ± 0.01 (A42) to 5.77 ± 0.02 (Q), and from 2.9 ± 0.1 (11) to 12.7 ± 0.4 (A42) ml of NaOH (0.1 N), respectively. The concentration of lactic and acetic acid ranged from 3.1 ± 0.2 (Q) to 30.4 ± 0.3 (A42) mmol/kg, and 0.9 ± 0.1 (Q) to 11.5 ± 0.3 (A7) mmol/kg, respectively. Overall, the samples that claimed to represent baker’s yeast breads (36 samples) exhibited higher pH values, ranging from 5.85 ± 0.04 (A14) to 6.40 ± 0.02 (19), and lower values of TTA from 1.0 ± 0.2 (A50) to 3.8 ± 0.2 (A30) ml of NaOH (0.1 N). Where detectable, lactate and acetate were present at concentrations lower than 2 mmol/kg. As expected, baker’s yeast breads that were chemically acidified contained pH and TTA levels of ca. 4.3 and 13.0 ml of NaOH (0.1 N), respectively.

### Primers and quantitative PCR (qPCR) optimization

Under the experimental conditions of this study, various primer pairs which had previously been designed to detect lactic acid bacteria^30–33^ did not allow the differentiation between sourdough and baker’s yeast breads (data not shown). In addition, some of these primer pairs^30, 31^ were not able to amplify *Leuconostoc* spp., which are significant components of sourdough microbiota.

Therefore, primers SK(F)/SK(R) were manually designed, according to the consensus sequence obtained by alignment of 16S rRNA-encoding gene sequences from members of various lactic acid bacterial genera (*Lactobacillus*, *Lactococcus*, *Leuconostoc*, *Weissella*, and *Pediococcus*) that are typically encountered in sourdoughs^34^ and the main flour contaminants (*Acinetobacter*, *Bacillus*, *Enterobacter*, *Pseudomonas*, *Sphingomonas*, *Staphylococcus*, *Rhizobium*, and *Erwinia*)^35–37^ (Table 1). To assess the specificity of this newly designed primer pair, standard PCR reactions were carried out using DNA extracted from 82 strains selected for the purpose of this study (Supplementary Table S2). As shown by agarose gel electrophoresis, the amplification procedure resulted for all (i.e. 59) tested lactic acid bacterial strains in the generation of a specific DNA product of ca. 178-bp (Supplementary Fig. S1), whereas no amplicon was generated when a DNA template was employed from the remaining 23 (non-lactic acid) bacterial strains that are frequently found to contaminate wheat flour (Supplementary Fig. S1).

**Table 1:** List of lactic acid bacteria and main flour contaminants used for consensus primer design.

Target	Genbank accession numbers for consensus primer design
Lactic acid bacteria	
*Weissella cibaria* DSM 15878	AJ295989
*Leuconostoc citreum* ATCC 49370	NR_041727.1
*Leuc*. *mesenteroides* ATCC 8293	KX886793.1
*Lactobacillus sanfranciscensis* DSM 20451	X76327
*Lactobacillus plantarum* WCFS1	KC429782.1
*Lactobacillus brevis* ATCC 14869	NR_044704.1
*Lactobacillus pentosus* ATCC 8041	KT025934.1
*Lactococcus lactis* NCDO 604	NR_040955.1
*Pediococcus acidilactici* DSM 20284	AJ305320
Flour contaminants	
*Acinetobacter johnsonii* DSM 6963	HE651920
*Bacillus pseudofirmus* DSM 8715	X76439.1
*Enterobacter cloacae* DSM 30054	HE978272
*Pseudomonas synxantha* DSM 18928	D84025.1
*Sphingomonas* sp. DSM 21917	FJ233842.1
*Staphylococcus aureus* DSM 20231	NR_037007.1
*Rhizobium fabae* DSM 19331	DQ835306
*Erwinia tracheiphila* DSM 21139	Y13250

Breads inoculated with various cell numbers (ca. 6.0–9.0 Log cfu/g) from a pure culture of *L*. *plantarum* type strain WCFS1 were subjected to DNA extraction, and qPCR was carried out. Cycle threshold (C_T_) values were plotted against the gene copy number and a linear calibration curve with a correlation coefficient (R^2^) of 0.9948, and linearity within 4 logarithmic cycles was obtained (Supplementary Table S3, Supplementary Fig. S2).

**Figure 1:**
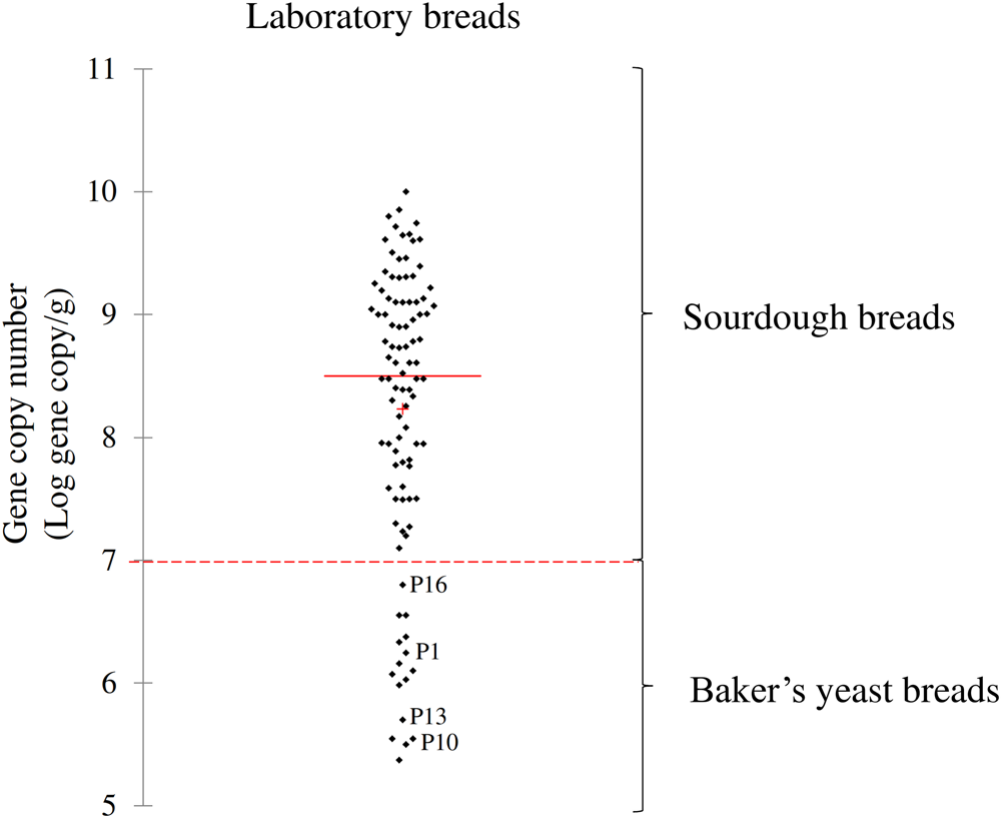
Scatterplot based on gene copy number as determined from laboratory breads. Scatterplot based on gene copy numbers of traditional and dried sourdough or baker’s yeast breads made under pilot scale/laboratory conditions. Mean (+), median (red line), and cut-off (dashed line) values are shown. Dried sourdough breads coded as P1, P10, P13, and P16 were made using 2.2% [wt/wt] of dried sourdough. The ingredients and technology parameters used for bread made at the pilot plant scale (laboratory) are reported in Table 2.

Several optimization efforts were made by varying primer and template concentrations (0.25–1 μl), annealing temperature (50–60 °C), number of cycles (30–40) and threshold baseline (0.1–0.8), ultimately resulting in a robust and reliable qPCR protocol that is able to differentiate sourdough from baker’s yeast breads. The optimization resulted in the use of 0.75 µl of 100 µM of each primer, an annealing temperature of 55 °C, and a PCR reaction consisting of 35 amplification cycles. In order to determine the C_T_ for each sample, the same baseline threshold at which sample fluorescence could be distinguished from background noise was always manually adjusted at 0.4. To define the detection limit that discriminated between sourdough and baker’s yeast breads, all laboratory sourdough breads, made with traditional sourdoughs and with various cell numbers of lactic acid bacteria, were used (Table 2). According to cell densities of sourdough breads (6.0 ± 0.2–8.7 ± 0.1 Log cfu/g), the gene copy number significantly (P < 0.05) varied (7.2 ± 0.3–9.9 ± 0.3 Log gene copy/g). When baker’s yeast breads (lactic acid bacteria of 2.5 ± 0.2 to 4.4 ± 0.3 Log cfu/g) were assessed, estimated gene copy number (Log gene copy/g) ranged from 5.4 ± 0.6 to 6.3 ± 0.2 (Supplementary Table S4 and Fig. 1). In order to partly simulate industrially sourdough bread production, which employs dried sourdough preparations or long term fermentation, 21 breads (coded as P1–P21) were manufactured under pilot plant conditions using commercial dried sourdoughs (Table 2). However the lactic acid bacterial cell density of these breads was lower than 4 Log cfu/g (Table 2), due to the drying process which the sourdough had been subjected to, and the lactic acid bacteria gene copy numbers varied in the assessed breads according to the percentage of dried sourdough used in the corresponding manufacturing process (Supplementary Table S4 and Fig. 1). In particular, the gene copy number varied from 5.5 ± 0.1 (P10) to 10.0 ± 0.3 (P6), whereas it was >7 Log gene copy/g when the breads were manufactured according to the recipe reported on the label of the dried sourdough packaging (4% [wt/wt of dough]) (Table 2 and Supplementary Table S4).

**Table 2:** Ingredients, technology parameters and cell density of lactic acid bacteria of traditional type I and dried sourdough or baker’s yeast breads made at the pilot plant scale.

Bread code	Flour (g)	Water (g)	Sourdough (g)	Lactic acid bacteria* (Log cfu/g)	Baker’s yeast (g)	Time of leavening (h)
Type I sourdough TA						
TA1	112.5	67.5	20	7.9 ± 0.1^bc,cd,de,ef^	3	1.5
TA2	100	60	40	8.2 ± 0.2^de,ef,fg,gh,hi^		
TA3	87.5	52.5	60	8.4 ± 0.1^fg,gh,hi,ij^		
TA4	75	45	80	8.5 ± 0.3^gh,hi,ij^		
TA5	100	60	40	8.2 ± 0.2^de,ef,fg,gh,hi^	–	4
TA6	87.5	52.5	60	8.4 ± 0.1^fg,gh,hi,ij^		
TA7	75	45	80	8.5 ± 0.3^gh,hi,ij^		
Type I sourdough TD						
TD1	112.5	67.5	20	8.1 ± 0.2^cd,de,ef,fg,gh^	3	1.5
TD2	100	60	40	8.3 ± 0.2^ef,fg,gh,hi,ij^		
TD3	87.5	52.5	60	8.5 ± 0.3^gh,hi,ij^		
TD4	75	45	80	8.7 ± 0.1^ij^		
TD5	100	60	40	8.3 ± 0.2^ef,fg,gh,hi,ij^	–	4
TD6	87.5	52.5	60	8.5 ± 0.3^gh,hi,ij^		
TD7	75	45	80	8.7 ± 0.1^ij^		
Type I sourdough MTA						
MTA1	112.5	67.5	20	7.7 ± 0.2^y,ab,bc,cd^	3	1.5
MTA2	100	60	40	8.2 ± 0.2^de,ef,fg,gh,hi^		
MTA3	87.5	52.5	60	8.3 ± 0.1^ef,fg,gh,hi,ij^		
MTA4	75	45	80	8.6 ± 0.3^hi,ij^		
MTA5	100	60	40	8.2 ± 0.2^de,ef,fg,gh,hi^	–	4
MTA6	87.5	52.5	60	8.3 ± 0.1^ef,fg,gh,hi,ij^		
MTA7	75	45	80	8.6 ± 0.3^hi,ij^		
Type I sourdough ALB						
ALB1	112.5	67.5	20	7.8 ± 0.3^ab,bc,cd,de^	3	1.5
ALB2	100	60	40	8.1 ± 0.3^cd,de,ef,fg,gh^		
ALB3	87.5	52.5	60	8.4 ± 0.1^fg,gh,hi,ij^		
ALB4	75	45	80	8.6 ± 0.2^hi,ij^		
ALB5	100	60	40	8.1 ± 0.3^cd,de,ef,fg,gh^	–	4
ALB6	87.5	52.5	60	8.4 ± 0.1^fg,gh,hi,ij^		
ALB7	75	45	80	8.6 ± 0.2^hi,ij^		
Type I sourdough CG						
CG1	112.5	67.5	20	7.7 ± 0.3^y,ab,bc,cd,de^	3	1.5
CG2	100	60	40	8.2 ± 0.3^de,ef,fg,gh,hi^		
CG3	87.5	52.5	60	8.5 ± 0.1^gh,hi,ij^		
CG4	75	45	80	8.5 ± 0.1^gh,hi,ij^		
CG5	100	60	40	8.2 ± 0.3^de,ef,fg,gh,hi^	–	4
CG6	87.5	52.5	60	8.5 ± 0.1^gh,hi,ij^		
CG7	75	45	80	8.5 ± 0.1^gh,hi,ij^		
Type I sourdough V						
V1	44.4	135.6	20	7.3 ± 0.1^t,w,x,y,z^	3	1.5
V2	83.3	66.7	50	7.6 ± 0.2^x,y,z,ab,bc^		
V3	55.5	44.4	100	7.8 ± 0.2^ab,bc,cd,de^	3	1.5
V4	27.7	22.3	150	7.9 ± 0.3^bc,cd,de,ef^		
V5	–	–	200	8.3 ± 0.1^ef,fg,gh,hi,ij^		
Type I sourdough V*d*						
V*d*1	44.4	135.6	20	6.0 ± 0.2^o^	3	1.5
V*d*2	83.3	66.7	50	6.5 ± 0.1^p^		
V*d*3	55.5	44.4	100	6.8 ± 0.3^p,q,r,s^		
V*d*4	27.7	22.3	150	6.9 ± 0.2^q,r,s,v^		
V*d*5	–	–	200	7.3 ± 0.1^t,u,v,w,x,y,z^		
Type I sourdough ALA						
ALA1	44.4	135.6	20	7.3 ± 0.1^t,u,w,x,y,z^	3	1.5
ALA2	83.3	66.7	50	7.6 ± 0.3^x,y,z,ab,bc^		
ALA3	55.5	44.4	100	7.8 ± 0.2^ab,bc,cd,de^		
ALA4	27.7	22.3	150	7.9 ± 0.1^bc,cd,de,ef^		
ALA5	–	–	200	8.3 ± 0.2^ef,fg,gh,hi,ij^		
Type I sourdough ALA*d*						
ALA*d*1	44.4	135.6	20	6.1 ± 0.2^o^	3	1.5
ALA*d*2	83.3	66.7	50	6.6 ± 0.2^p,q,r^		
ALA*d*3	55.5	44.4	100	6.9 ± 0.3^q,s,t,u,v^		
ALA*d*4	27.7	22.3	150	7.00 ± 0.1^s,t,u,v,w^		
ALA*d*5	–	–	200	7.2 ± 0.2^s,t,u,v,w,x^		
Type I sourdough BA						
BA1	44.4	135.6	20	7.4 ± 0.2^x,y,z,ab^	3	1.5
BA2	83.3	66.7	50	7.6 ± 0.1^x,y,z,ab,bc^		
BA3	55.5	44.4	100	7.8 ± 0.2^ab,bc,cd,de^		
BA4	27.7	22.3	150	8.0 ± 0.1^bc,cd,de,ef,fg^		
BA5	–	–	200	8.4 ± 0.3^fg,gh,hi,ij^		
Type I sourdough BA*d*						
BA*d*1	44.4	135.6	20	6.0 ± 0.3^o^	3	1.5
BA*d*2	83.3	66.7	50	6.5 ± 0.1^p,r^		
BA*d*3	55.5	44.4	100	6.9 ± 0.3^q,s,u,v^		
BA*d*4	27.7	22.3	150	7.1 ± 0.2^s,t,u,v,w^		
BA*d*5	–	–	200	7.3 ± 0.1^t,u,v,w,x,z^		
Baker’s yeast bread						
BY1	111.1^a^	88.9	–	3.1 ± 0.1^i,j^	4	1.5
BY2				3.0 ± 0.3^h,i,j^		
BY3				3.0 ± 0.1^h,i^		
BY4				2.5 ± 0.3^e,f,g^		
BY5				2.5 ± 0.2^f,g^		
BY6				3.7 ± 0.2^m^		
BY7	111.1^b^	88.9	–	3.1 ± 0.1^h,i,j^	4	1.5
BY8				4.4 ± 0.3^n^		
BY9				2.6 ± 0.2^f,g^		
BY10				3.7 ± 0.3^m^		
BY11	111.1^b^	88.9	–	3.5 ± 0.1^k,m^	4	1.5
BY12				4.4 ± 0.1^n^		
Dried sourdough brand A						
P1	106.7	88.9	4.4	3.4 ± 0.1^j,k,l,m^	–	6
P2	102.2	88.9	8.9	3.1 ± 0.4^i,j,l^		
P3	71.1	88.9	40.0	3.7 ± 0.2^m^		
Dried sourdough brand B						
P4	106.7	88.9	4.4	2.5 ± 0.2^e,f,g^	–	6
P5	102.2	88.9	8.9	2.4 ± 0.4^e,f,g^		
P6	71.1	88.9	40.0	2.3 ± 0.2^e,f^		
Dried sourdough brand C						
P7	106.7	88.9	4.4	1.5 ± 0.1^a,b^	–	6
P8	102.2	88.9	8.9	1.7 ± 0.2^b,c^		
P9	71.1	88.9	40.0	1.8 ± 0.2^b,c,d^		
Dried sourdough brand D						
P10	106.7	88.9	4.4	2.8 ± 0.4^g,h,i^	–	6
P11	102.2	88.9	8.9	2.5 ± 0.3^f,g^		
P12	71.1	88.9	40.0	2.5 ± 0.1^f,g^		
Dried sourdough brand E						
P13	106.7	88.9	4.4	1.9 ± 0.1^c,d^	–	6
P14	102.2	88.9	8.9	2.1 ± 0.2^d,e^		
P15	71.1	88.9	40.0	1.8 ± 0.1^b,c,d^		
Dried sourdough brand F						
P16	106.7	88.9	4.4	1.2 ± 0.2^a^	–	6
P17	102.2	88.9	8.9	1.5 ± 0.2^a,b^		
P18	71.1	88.9	40.0	1.3 ± 0.4^a^		
Dried sourdough brand G						
P19	106.7	88.9	4.4	3.2 ± 0.2^i,j,k,l^	–	6
P20	102.2	88.9	8.9	3.5 ± 0.1^k,l,m^		
P21	71.1	88.9	40.0	2.7 ± 0.4^f,g,h^		

^a^ *Triticum aestivum*; ^b^ *Triticum durum*.

*Cell density of lactic acid bacteria of the sourdough or baker’s yeast breads before the dough was subjected to baking, as estimated by plating on agar media at 30 or 37 °C for 48 h under anaerobiosis. Values are means ± standard deviation of three batches analysed in triplicate (n = 9). Values within a column with different superscript single and/or pairs letters are significantly different (P < 0.05).

As shown from the scatterplot based on gene copy numbers, 96.4% of the laboratory sourdough breads were scattered above 7 Log gene copy/g. The remaining 3.6% was represented by four dried sourdough breads (coded as P1, P10, P13, and P16) made using 2.2% [wt/wt of dough] of dried sourdough, unlike from the manufacturer’s suggestion (4% [wt/wt of dough]). However, 100% of baker’s yeast breads were scattered below a gene copy number of 7 Log gene copy/g (Fig. 1). The chemically acidified baker’s yeast breads were determined to contain gene copy numbers <7 Log gene copy/g.

### Discrimination between bakery sourdough and baker’s yeast breads

**Figure 2:**
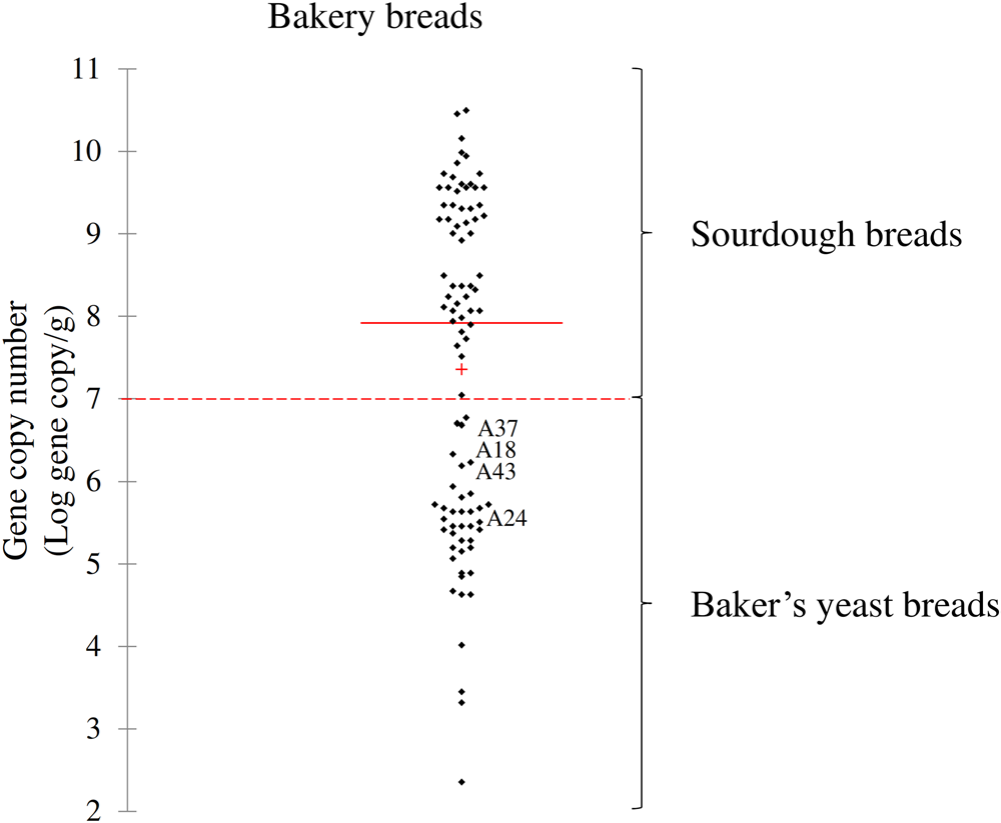
Scatterplot based on gene copy number obtained from bakery breads. Scatterplot based on gene copy numbers of sourdough or baker’s yeast breads collected from various bakeries. Mean (+), median (red line), and cut-off (dashed line) values are shown. Breads coded as A18, A24, A37, and A43 were claimed as sourdough breads. Information on the commercial breads collected from bakeries is reported in Supplementary Table S6.

The efficiency of the optimized qPCR protocol was further assessed on 93 breads, which had been collected from bakeries (Supplementary Table S5 and Fig. 2). Ninety-three per cent of samples claimed as sourdough breads were scattered above the gene copy number of 7 Log gene copy/g, whereas 100% of those sold as baker’s yeast breads were grouped below the value of 7 Log gene copy/g. The exceptions were the bakery sourdough breads coded as A18, A24, A37, and A43, which showed gene copy numbers of 6.2 ± 0.8, 5.5 ± 0.5, 6.7 ± 0.6, and 6.2 ± 0.5 Log gene copy/g, respectively. The biochemical features of these breads were pH 5.75 ± 0.01–5.01 ± 0.06, TTA 5.0 ± 0.3–3.0 ± 0.1 ml of NaOH 0.1 N, and 7.2 ± 0.1–5.1 ± 0.2 and 3.5 ± 0.4–0.5 ± 0.2 mmol/kg of lactate and acetate (Supplementary Table S1).

**Figure 3:**
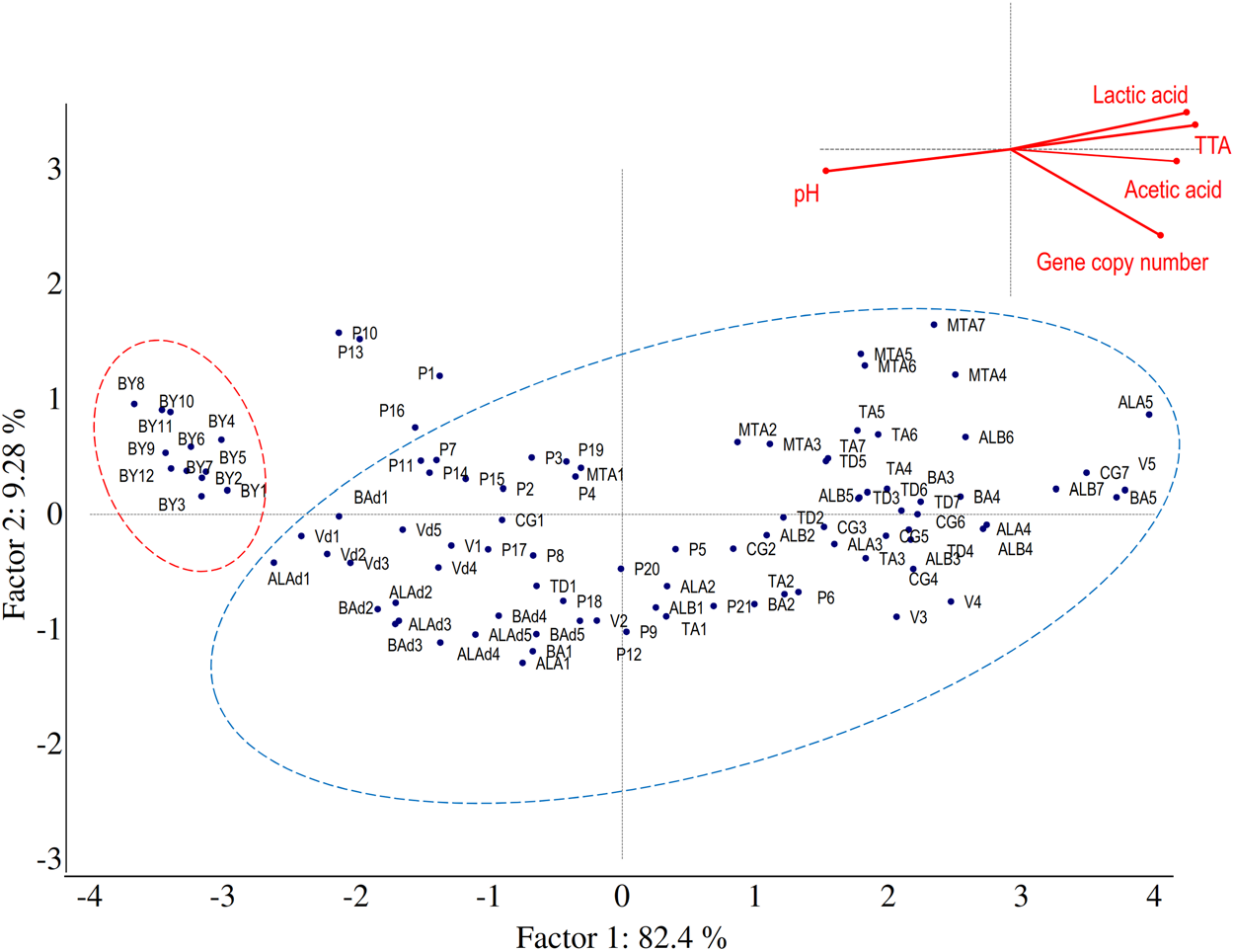
Principal Component Analysis. Score and loading plots of first and second principal components after principal component analysis based on values of gene copy, pH, TTA (ml NaOH 0.1 N) and concentration of lactic and acetic acids (mmol/kg) of breads made under pilot plant conditions (laboratory). Traditional and dried sourdough and baker’s yeast breads are delineated by blue and red line ovals, respectively. The ingredients and technology parameters used for bread made at the pilot plant scale (laboratory) are reported in Table 2.

**Figure 4:**
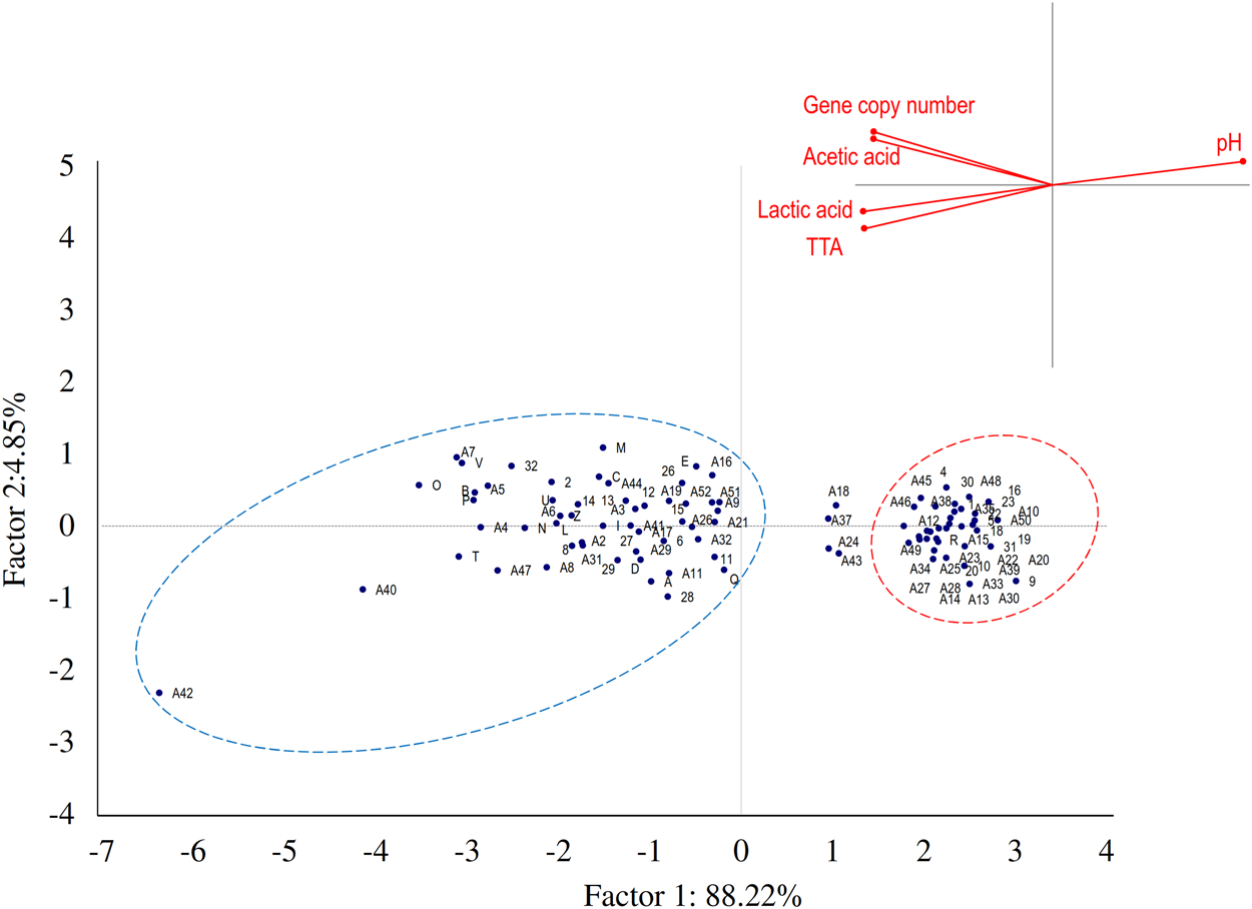
Principal Component Analysis. Score and loading plots of first and second principal components after principal component analysis based on values of gene copy, pH, TTA (ml NaOH 0.1 N) and concentration of lactic and acetic acids (mmol/kg) of breads collected from bakeries. Sourdough and baker’s yeast breads are delimited by blue and red line circles, respectively. Information on the commercial breads collected from bakeries is reported in Supplementary Table S6.

Values of gene copy (Log gene copy/g), pH, TTA, and lactic and acetic acid concentrations of laboratory sourdough and baker’s yeast breads were assessed by means of a Principal Component Analysis (PCA) (Fig. 3). Two significant PCs explained 82.40% (PC1) and 9.28% (PC2) of the total variance of the data. Baker’s yeast breads were clustered in the same zone of the plane (red line oval), which represents the lowest values of gene copy (<7 Log gene copy/g) and pH (5.99–6.25), and the lowest TTA values (0.6–1.7 ml of NaOH 0.1 N), and concentration of organic acids (<1.0 mmol/kg). When the gene copy number values increased to more than 7 Log gene copy/g, sourdough breads were clearly separated in another zone of the plane (blue line oval). The largest dispersion of these samples was mainly due to pH variation (pH values ranging between 3.93 and 5.69), TTA (3.0–13.4 ml of NaOH 0.1 N) and lactic and acetic acid concentrations (4.7–28.8 and 2.3–11.7 mmol/kg, respectively). Sourdough breads with both the highest values of gene copy number and the highest acidity levels were scattered from the central to the edge of the right zone of the plane. Dried sourdough breads coded as P1, P10, P13, and P16 did not cluster with either baker’s yeast or sourdough breads. This dispersion was probably due to the values of gene copy number being lower than 7 Log gene copy/g, which moved the samples close to the baker’s yeast bread cluster, and the biochemical features being similar to sourdough breads which, in contrast, positioned the samples close to sourdough breads cluster. Even more obvious was the differentiation between bakery sourdough and baker’s yeast breads (Fig. 4). The two PCs explained 93.1% of the total data variance. Baker’s yeast breads (red line oval) were separated on the right zone of the plot (values of gene copy <7 Log gene copy/g, pH 5.85–6.40, TTA 1.0–3.2 ml of NaOH (0.1 N) and concentration of organic acids <2 mmol/kg). As the values for gene copy number increased (>7 Log gene copy/g), sourdough breads (blue line oval) were grouped in the left part of the plane. Within this group, Q and A42 breads represented the endpoints, which showed the highest and lowest values of pH, respectively. Breads coded as A18, A24, A37, and A43, which were claimed to be sourdough breads were scattered between baker’s yeast and sourdough breads. When both types of breads, made under pilot plant conditions (laboratory) or collected from bakeries, were analysed by PCA, the two PCs explained ca. 91% of the total data variance (data not shown). Regardless of the manufacture location, sourdough samples were distinctly distributed from baker’s yeast breads, which confirmed the previous results (Figs 3 and 4).

As expected, the Pearson correlations of pH with TTA (−0.953) and concentrations of lactate (−0.919) and acetate (−0.817) of all examined (191) breads were very strong. Although strong, the correlation between pH and gene copy number was lower (0.730) (Table 3).

**Table 3:** Pearson correlation matrix between the gene copy number (Log gene copy/g) and the biochemical characteristics of laboratory and bakery sourdough breads.

	Gene copy number	pH	TTA	Lactic acid	Acetic acid
Gene copy number	**1**	−0.730	0.701	0.708	0.717
pH	−0.730	**1**	−**0**.**953**	−**0**.**919**	−0.817
TTA	0.701	−**0**.**953**	**1**	**0**.**932**	0.795
Lactic acid	0.708	−**0**.**919**	**0**.**932**	**1**	0.746
Acetic acid	0.717	−0.817	0.795	0.746	**1**

TTA, total titratable acidity.

Boldface values (lower triangular matrix) represent highly correlated descriptors with values >0.9 or <−0.9.

## Discussion

To declare sourdough as the leavening agent of bread without actually using it constitutes a fraudulous act of consumer deception. In contrast, the use of sourdough results in a traditional, superior quality bread with a premium market price, and meets consumer expectations. All European breads that enjoy the status of Protected Designation of Origin (Pane di Altamura and Pagnotta del Dittaino) or of Protected Geographical Indication (Pane di Matera, Coppia Ferrarese and Pane Casareccio di Genzano) are manufactured with sourdough. Except for France, no other national legislation regulates and protects the use of sourdough in bread making, also because a reliable policing methodology is currently lacking. In order to fill this technological hiatus we developed a Quantitative PCR (qPCR) method^38^ to discriminate between breads made with and without sourdough.

qPCR is used in food sciences to uncover ingredient fraud^23, 24, 39^ or to detect specific ingredients^40^, allergens^41^ or gluten^42^. It is also a powerful method to detect and quantify food microbial populations for safety^26, 43, 44^, spoilage^43^ and processing (starters or probiotics) purposes^45–47^. The qPCR method described in the current study is aimed at distinguishing sourdough from the other leavened breads by detecting the very complex and diverse lactic acid bacterial microbiota^34^. For this purpose, qPCR did not consider specific primers targeting selected genes (e.g., *tuf* and *invA*)^48, 49^, but used universal primers for lactic acid bacteria, which were designed based on the 16S rRNA-encoding gene^30–33^. The primer specificity was assessed towards 59 strains (representing 38 bacterial species), which belong to the genera of (sourdough-associated) lactic acid bacteria^34^, in addition to 23 bacterial species that are considered flour contaminants^35–37^. A common ca. 178-bp 16S rRNA gene fragment was amplified only when the DNA of sourdough lactic acid bacteria was used. The degenerate primer pair was shown to exclude some of the most commonly encountered flour contaminants, such as *Acinetobacter*, *Bacillus*, *Enterobacter*, *Pseudomonas*, *Sphingomonas*, *Staphylococcus*, *Rhizobium*, *Pantoea*, *Delftia*, *Commamonas*, *Serratia*, *Atlantibacter* and *Erwinia* ^35–37^. The short length of the amplicon was selected to counter a food processing issue, caused by the fact that heating (e.g., baking)^50, 51^ and acidification (e.g., most of the sourdough breads)^52, 53^ may result in DNA fragmentation^54^. DNA degradation was observed in dried sourdoughs, possibly due to DNA depurination by low pH^29^. Previously, primers targeting the 16S rRNA gene were used for quantitative detection of sourdough lactic acid bacteria during sorghum sourdough fermentation^28^ or to characterize dried sourdoughs^29^. The former report^28^ describes the simultaneous quantification of four genotypes though it was not suitable for more complex and/or undefined sourdough microbiotas such as those encountered in the current study. In the latter report^29^, the amplification of a 341-bp 16S rRNA gene fragment allowed the detection of *Lactobacillus*, *Leuconostoc*, *Pediococcus* and *Weissella* species. The use of the same primers failed in this study, possibly because either the calibration curve was based on pure cultures^29^ or the total DNA directly extracted from breads was used rather than pure cultures. To efficiently apply qPCR in food diagnostics, calibration curves, which associate gene copy number values (Log gene copy/g) with C_T_ values, have to be created in food matrices^44^. The co-extraction of inhibitory compounds from food matrices has to be considered as they may interference with qPCR reactions. Therefore, a calibration curve was initially constructed using sourdough breads inoculated with pure cultures of *L*. *plantarum*, resulting in a linear calibration curve with a correlation coefficient (R^2^) of ≥0.99. The presence of multiple copies of the 16S rRNA gene per genome was taken into consideration according to the complexity and diversity of the sourdough lactic acid bacteria microbiota species/strains. Nevertheless, gene copy number related to baker’s yeast breads suffered from the limitation of being outside the calibration curve and therefore no solid conclusions may be drawn from such data other than that lactic acid bacteria are present at low numbers which are therefore not a reflection of industrial conditions^4, 15^.

The qPCR method of this study did not intend to quantify certain species but, rather, aimed at discriminating between breads fermented with relevant numbers of lactic acid bacteria (sourdough) and those started with leavening agents (e.g., baker’s yeast), where these bacteria are absent or inadvertent contaminants. Sourdough breads were made under pilot scale conditions to cover the whole potential range of lactic acid bacterial cell densities, even though the lowest values (e.g., ca. 6.0–6.1 Log cfu/g) are rather far removed from those (ca. 7.0–9.0 Log cfu/g) typically found in sourdough bakery breads^4^. A conspicuous number of the industrially produced sourdough bread employs dried or pasteurized sourdough preparations^18^ or long term fermentation^55^ where the lactic acid bacteria are dead and lysed before the bread dough is mixed and their DNA may thus be subject to degradation at the dough stage or (early) during baking. This may prevent detection of DNA of lactic acid bacteria from bread. Based on the above considerations, 21 dried sourdough breads were manufactured at pilot plant scale employing different amounts of commercial dried sourdoughs in the recipes. All laboratory breads made with traditional type I sourdoughs generated gene copy number values of >7 Log gene copy/g, while all laboratory baker’s yeast breads clustered below a gene copy number of 7 Log gene copy/g. The application of the qPCR to dried sourdoughs breads corroborated the above results, generating gene copy number values of >7 Log gene copy/g when the dried sourdough was used according or exceeding (fivefold) to the manufacturer’s suggestions (4 or 20% [wt/wt of dough]). Four out of seven dried sourdough breads, made using half of the suggested amount of dried sourdoughs, corresponding to the gene copy number values <7 Log gene copy/g were scattered out of the sourdough bread cluster. These findings highlight the strict correlation between the amount of lactic acid bacteria DNA in the bread and the gene copy number. Similar results were obtained when the robustness of the method was assessed on 93 commercial breads that had been produced with or without sourdough. Approximately 96% of the generated results were consistent with the manufacture process as stated on the label of the breads. The remaining 4%, bread coded as A18, A24, A37, and A43, which generated gene copy number values of <7 Log gene copy/g, were sold as sourdough breads from artisanal bakeries but were not provided with any stated manufacturing details. The biochemical features of such breads were close to those made at laboratory level, harboring a low lactic acid bacterial cell density. Therefore, it cannot excluded that those breads had been manufactured with either low sourdough amount or sourdough harboring lactic acid bacterial cell numbers below those produced under industrial conditions^4, 15, 16, 56, 57^.

As shown by the correlation analysis, discriminatory gene copy values were shown to be weakly related to the values of pH and TTA, and the concentration of lactic and acetic acids, while the correlation between pH and gene copy number (Log gene copy/g) was lower (0.730). The combination of the qPCR results and those of the Pearson’s correlation, confirmed that such indirect indices (e.g. pH and TTA) are not sufficiently powerful to reliably identify sourdough breads. They may, where appropriate, support the information of a more reliable method, though one has to keep in mind that acidification may be achieved by the addition of organic acids rather than by biological means.

The use of sourdough has to be legally protected and a reliable method for its authentication was provided. Frauds such as declaring sourdough fermentation, but using other leavening agents, can be detected by our method.

## Materials and Methods

### Bacteria and growth condition

The 59 strains of lactic acid bacteria and 23 strains representing common flour contaminants used in this study are listed in Supplementary Table S2. Strains of lactic acid bacteria were previously isolated from sourdoughs (except for certain strains that were obtained from the ATCC or DSM strain collections) and identified by partial sequencing of the 16S rRNA, *recA*, *pheS*, and *rpoA* genes. *Lactobacillus fermentum*, *Lactobacillus pentosus*, *Lactobacillus amylovorus*, *Lactobacillus plantarum*, *Lactobacillus hilgardii*, *Lactobacillus hammesii*, *Lactobacillus farciminis*, *Leuonostoc citreum*, *Weissella confusa*, and *Weissella cibaria* strains were cultured on modified MRS (mMRS) medium, containing 1% [wt/vol] maltose, 5% [vol/vol] fresh yeast extract, and pH 5.6, at 30 °C for 24 h. *Lactobacillus curvatus*, *Lactobacillus brevis*, *Leuconostoc mesenteroides* subsp. *cremoris*, *Leuc*. *mesenteroides* subsp. *dextranicum*, *Leuc*. *mesenteroides* subsp. *mesenteroides*, *Lactobacillus alimentarius*, *Lactobacillus casei*, *Lactobacillus paralimentarius*, *Lactobacillus acidophilus*, *Lactobacillus kunkeei*, *Pediococcus pentosaceus*, and *Pediococcus acidilactici* strains at 30 °C, and *Lactobacillus delbrueckii*, *Lactobacillus helveticus*, *Lactobacillus reuteri*, *Enterococcus faecalis*, and *Enterococcus faecium* strains at 37 °C were grown for 24 h on MRS (Oxoid, Basingstoke, Hampshire, United Kingdom). *Lactobacillus rossiae*, and *Lactobacillus sanfranciscensis* strains were cultivated on sour dough bacteria media (SDB)^58^ at 30 °C for 24 h, and *Lactococcus lactis* strain on M17 containing 0.5% [wt/vol] lactose (Oxoid) at 37 °C for 24 h. *Lactobacillus acidifarinae* and *Lactobacillus amylolyticus* were grown for 24 h on MRS (Oxoid) supplemented with 0.05% [wt/vol] cysteine-hydrochloride (Sigma-Aldrich, St. Louis, MO, USA) and pH 5.2 at 30 and 45 °C, respectively. *Lactobacillus nantensis* on MRS (Oxoid) supplemented with 0.05% [wt/vol] cysteine-hydrochloride (Sigma-Aldrich), 1% [wt/vol] maltose (Oxoid), and 0.5% [vol/vol] fresh yeast extract at 30 °C for 24 h. *Lactobacillus frumenti* on Medium 638 (www.dsmz.de/catalogues/catalogue-microorganisms.html) with pH 5.4, at 40 °C for 24 h. *Lactobacillus namurensis* was cultivated for 24 h on medium 231 (www.dsmz.de/) supplemented with 0.7% [wt/vol] maltose (Oxoid) and rye-bran extract at 30 °C. *Lactobacillus pontis* was grown on medium 638 (www.dsmz.de/) at 30 °C for 24 h. *Lactobacillus panis* on medium 859 (www.dsmz.de/) at 37 °C for 24 h. *Lactobacillus crispatus* was grown for 48 h on medium 58 (www.dsmz.de/) at 37 °C, under anaerobic conditions. *L*. *plantarum WCFS*1 type strain was also used in the assessment of the primer specificity. It was grown in MRS (Oxoid) at 37 °C for 24 h. Strains of flour contaminants were previously isolated from flours or wheat plants. *Serratia marcescens*, *Pseudomonas fluorescent*, *Enterobacter* sp., *Pseudomans* sp., *Delftia* sp., *Rhizobium* sp., *Commamonas* sp., *Sphingomonas* sp., *Erwinia* sp., and *Staphylococcus* sp. were grown on Nutrient broth (Oxoid) at 25 °C for 24 h. *Staphylococcus capitis* and *Atlantibacter hermannii* were cultivated for 24 h at 37 °C on MRS and Nutrient broth (Oxoid), respectively. *Bacillus megaterium* and *Bacillus* sp., were cultivated for 24 h at 30 °C on Luria-Bertani medium (Oxoid). *Pantoea agglomerans* and *Pantoea* sp. were grown for 24 h at 30 °C on Dextrose tryptone broth (Oxoid). *Enterobacter aurogenes*, *Acinetobacter calcoaceticus*, and *Acinetobacter* sp. were grown on Brain-heart infusion medium (BHI) for 24 h at 25 °C. The same culture media used for strain isolation were used for cultivation. Cultures were maintained as stocks in 15% [vol/vol] glycerol at −80 °C and routinely propagated at 30, 37, 40 or 45 °C for 24 h in the respective culture media.

### Traditional type I sourdough preparation and enumeration of lactic acid bacteria

*Triticum durum* (TD) and *Triticum aestivum* (TA) flours were used to prepare laboratory type I sourdoughs. In detail, 166.7 g of flour (TD or TA) and 133.3 ml of tap water were used to make 300 g of dough (dough yield [dough weight × 100/flour weight], of 180) with a continuous high-speed mixer (60 × g, dough mixing time of 5 min) (Chopin & Co., Boulogne, Seine, France). Sourdough propagation was according to traditional back slopping protocols^4^, without using starter cultures or baker’s yeast. Daily, each sourdough was subjected to fermentation at 25 °C for 5 h, with the exception of the first propagation, which lasted for 8 h^35^. The propagation was carried out using 25% [wt/wt of dough] of the previous fermented dough to inoculate a fresh mixture of flour and tap water (DY of 180). Sourdoughs were propagated for 11 days, until the biochemical stability (pH of 4.24 ± 0.03–4.31 ± 0.02 and total titratable acidity, TTA of 8.4 ± 0.1–9.2 ± 0.2) and lactic acid bacteria cell density of ca. 8.9 ± 0.1–9.1 ± 0.4 Log cfu/g were achieved.

Traditional type I sourdoughs (DY of 180) from six artisan bakeries, which are located in the Southern of Italy, were also collected. MTA (Matera, Basilicata), ALA and ALB (Altamura, Apulia) were made with *T*. *durum* flour, and CG (Castellana Grotte, Apulia), V (Vico del Gargano, Apulia) and BA (Bari, Apulia) were made using *T*. *aestivum* flour. All these sourdoughs had cell densities of lactic acid bacteria ranging from ca. 8.9 ± 0.1 to 8.3 ± 0.3 Log cfu/g. To obtain cell numbers that ranged from 7.1 ± 0.2 to 7.3 ± 0.3 Log cfu/g, V, ALA and BA sourdoughs were diluted 1:10 with flour and water (DY of 180). These sourdoughs were indicated as V*d*, ALA*d* and BA*d*.

Sourdoughs were cooled down to 4 °C and analysed or used within 2 h after propagation or collection. All analyses were carried out in triplicate for each batch of sourdough. All sourdoughs were used as leavening agent under pilot plant-scale bread-making process conditions.

Ten gram of each traditional type I sourdoughs and bread dough after the fermentation was homogenized with 90 ml of sterile peptone water (0.1% [wt/vol] peptone, 0.85% [wt/vol] of NaCl) and serially diluted. Lactic acid bacteria were counted using the respective agar media, which were supplemented with cycloheximide (0.1 g/liter). Plates were incubated under anaerobioc conditions (AnaeroGen and AnaeroJar, Oxoid, Basingstoke, Hampshire, UK) at 30 or 37 °C for 48 h.

### Bread making and bread collection

Sourdough or baker’s yeast breads were either made at the pilot plant of the Department of Soil, Plant and Food Sciences (University of Bari Aldo Moro, Bari, Italy) or collected from bakeries.

Table 2 summarizes the ingredients, technology parameters and cell density of lactic acid bacteria of sourdough or baker’s yeast breads made at the pilot plant scale. The lactic acid bacterial cell density was enumerated at the end of the fermentation. Sourdough breads were made using either laboratory sourdoughs (TA and TD), or those collected from artisan bakeries (MTA, ALB, ALA, CG, V, and BA, and corresponding diluted samples). Various percentages (10 to 100% [wt/wt of dough]) of sourdoughs were used, and the fermentation duration ranged from 1.5 to 4 h. Fermentation times were selected on the basis of previous tests suggesting that 1.5 and 4 h were required to increase the dough volume when the baker’s yeast (1.5% [wt/wt of dough]) was or was not added to the dough, respectively. These conditions facilitate lactic acid bacterial cell densities to reach levels that ranged from 6.0 ± 0.2 to 8.7 ± 0.1 Log cfu/g before the dough was subjected to baking (Table 2). Baker’s yeast breads were also made, using various commercial preparations (2.0% [wt/wt of dough]). In this case, the formula did not provide the addition of sourdough, and lactic acid bacteria before baking ranged from 2.5 ± 0.2–4.4 ± 0.1 Log cfu/g. Two baker’s yeast breads were also chemically acidified with a mixture of lactic and acetic acid to achieve a pH of 4.0 before leavening. All breads made at the pilot plant had a DY of 180 and were baked at 200 °C for 30 min (Combo 3, Zucchelli, Verona, Italy).

Fifty-seven sourdough and 36 baker’s yeast breads were collected from Italian and Irish bakeries (Supplementary Table S6). The distinction between leavening agents (sourdough or baker’s yeast) was based on claims as given by bakers or as reported on the label information supplied by manufacturers.

In order to simulate the industrially produced dried sourdough breads^18^, 21 breads were manufactured at pilot plant scale employing seven commercial dried sourdoughs (Brand A–Brand G). In particular, each dried sourdough was used at 4% [wt/wt of dough], according to the recipes suggested by the manufactures as reported on the dried sourdough packaging label. In order to evaluate the sensitivity of the qPCR method, two different percentages (2.2% and 20% [wt/wt of dough]) of dried sourdoughs were used (Table 2). All dried sourdough breads were subjected to a 6 h fermentation, selected according to the time required by the doughs containing the lowest amount of dried sourdough (2.2% [wt/wt]) to double their volume.

The majority of these breads were made employing wheat flour, although a small number were produced using other cereals.

All breads were stored at −20 °C until further analysis.

### Chemical characterization of sourdoughs and breads

pH values were determined by a pH-meter (Model 507, Crison, Milan, Italy) with the aid of a food penetration probe. TTA was measured on 10 g of sourdough or bread samples, which were homogenized with 90 ml of distilled water for 3 min in a Bag Mixer 400P (Interscience, St Nom, France), and expressed as the amount (in ml) of 0.1 N NaOH to achieve pH 8.3.

Lactate and acetate concentrations were determined in the water-soluble extract of the breads. In particular, 4.8 g of bread was homogenized with 12 ml of Tris-HCl (50 mM, pH 8.8) buffer. Following incubation (60 min at 4 °C, under stirring), the resulting suspension was centrifuged (12,857 × *g*, 10 min, 4 °C). The supernatant was incubated overnight at 4 °C with perchloric acid [5% v/v] in 1:1 ratio and analysed using an ÄKTA Purifier™ system (GE Healthcare Bio-Sciences, Uppsala, Sweden), equipped with a refractive index detector (Perkin Elmer Corp., Waltham, MA), after centrifugation (12,857 × *g*, 10 min, 4 °C).

### Extraction of DNA from pure cultures and breads

Genomic DNA from pure cultures of bacterial strains was extracted using a DNeasy blood and tissue kit (Qiagen, SA, Courtaboeuf, France), according to the manufacturer’s instructions^59^. Total DNA extraction from breads was carried out using the Wizard Magnetic DNA Purification System for Food (Promega, Madison WI), following the manufacturer’s instructions. Each DNA extraction was performed in triplicate.

### Primer design and specificity assessment by PCR

Initially, several previously designed primer combinations and developed protocols^30–33^ were used to amplify (through qPCR) total DNA of laboratory baker’s yeast and sourdough breads, in order to evaluate their ability to discriminate between the two bread categories (Supplementary Table S7). 16S rRNA gene sequences of several bacterial type strains genera from GenBank database (http://www.ncbi.nlm.nih.gov) were aligned, using multiple sequence alignment software ClustalW^60^. Sequences corresponding to lactic acid bacteria that are commonly isolated from sourdough, belonging to *Lactobacillus*, *Leuconostoc*, *Weissella*, *Lactococcus*, and *Pediococcus* genera^35^, and to the most frequently encountered flour contaminants, belonging to the *Acinetobacter*, *Bacillus*, *Enterobacter*, *Pseudomonas*, *Erwinia*, *Staphylococcus*, *Rhizobium*, and *Sphingomonas* genera^35–37^. Based on the consensus of these multiple alignments, degenerated primers SKfw (5′-GGGGATAACAYYTGGAAACAG-3′) and SKrw (5′-CTCGGCTACGTATCATTGTCTTG-3′) were designed within a DNA region exhibiting high homology among lactic acid bacteria, while at the same time excluding the other genera (Table 1). Primer combinations were then assessed for dimer formation and template hairpins using Oligonucleotide Properties Calculator software (available at http://www.basic.northwestern.edu/biotools/oligocalc.html). The single-stranded oligonucleotide primers used in this study were synthesized by Eurofins (Ebersberg, Germany) and designed to amplify a DNA fragment of ca. 178 bp in length. First, primer specificity was checked *in silico* by jPCR (FastPCR online), after which the capacity to amplify targeted species was assessed using 59 strains of lactic acid bacteria and 23 strains that are frequently isolated as flour contaminants (Supplementary Table S2). PCR assays for primer specificity assessment were carried out in a 25 µl final volume, containing 12.5 µl Master Mix (Qiagen, Italy), RNase free water, 0.75 µl of each primer (100 µM) and DNA template at a final concentration of 100 ng/µl (in the final PCR mixture). PCRs were performed according to the Taq manufacturer’s instructions (which recommend a final template concentration of <250 ng/µl). The PCR core program was as follows: 95 °C for 10 min, followed by 35 cycles of 95 °C for 10 s, 55 °C for 30 s and 72 °C for 30 s and 72 °C for 7 min. Five microliters of the product was analysed by standard agarose gel electrophoresis (2% wt/vol).

### qPCR and calibration curve

Each qPCR reaction was performed in triplicate, using the Rotor-Gene SYBR Green RT-PCR Kit (Qiagen, Italy). The total reaction mixture (25 µl) contained 12.5 µl of SYBR Green Mix, 0.75 µl of 100 µM of each primer, 11 µl of RNase free water, and 1 µl of template. Primer efficiency was assessed using the same protocol, the amount of each primer (100 µM) in the range of 0.25–1 µl was only changed. Annealing temperature in the range 50–60 °C, number of cycles from 30 to 40, and threshold of 0.1 to 0.8 were assessed to optimize the qPCR protocol. Samples were processed by the Rotor-Gene 6000 (Corbett Research Ltd., Australia), according to the following thermal cycling program: 95 °C for 10 min, followed by 35 cycles of 95 °C for 10 s, 55 °C for 30 s and 72 °C for 30 s. Melt curve analysis of PCR amplicons was initiated at 60 °C, increasing by 1 °C until the final temperature of 95 °C was reached. Overall, qPCR consisted in a succession of amplification cycles having as results an exponential increase of amplicons (amplification products) that, in contrast with end-point PCR, can be monitored at every cycle (in real time) using a fluorescent reporter. The increase in fluorescence was plotted against the cycle number to generate the amplification curve, from which a quantification cycle (Cq) or cycle threshold (C_T_) value was determined. The C_T_ value corresponds to the number of cycles for which the amount of fluorescence (hence, of template) was significantly higher than the background fluorescence. Rotor-Gene 6000 Series Software, version1.7, facilitated the execution of the PCR program and data collection. C_T_ values for the examined breads were determined following manual adjustment of the threshold at the same value of 0.4. To set up a quantification method and to simulate the bread matrix effects, a calibration curve for qPCR was generated using breads inoculated with known cell densities (from 5.8 ± 0.2 to 9.8 ± 0.3 Log cfu/g) of a pure culture of *L*. *plantarum* type strain WCFS1 (Supplementary Table S3). DNA extracted from breads inoculated with *L*. *plantarum* WCFS1 was subjected to qPCR and the gene copy number (Log gene copy/g) was plotted against the C_T_ values (threshold of 0.4).

### Statistical analysis

Analyses were carried out on three independent replicates of each sample. Each replicate was analysed three times. Obtained data sets were subjected to one-way ANOVA; pair-comparison of treatment means was obtained by Tukey’s procedure at P < 0.05, using the statistical software Statistica 12.0 (StatSoft Inc., Tulsa, USA). Data sets related to C_T_, pH, TTA, and organic acids, were analysed through Principal Component Analysis (PCA), using the software XLSTAT 2016. The same data sets were subjected to Pearson correlation function by the use of Microsoft Xlstat software (Version 2014.5.03).

## Electronic supplementary material

Supplementary Information
