# Supplementary material for: Sourdough authentication: quantitative PCR to detect the lactic acid bacterial microbiota in breads
Source: Sci Rep. 2017 Apr 3;7:624. doi: 10.1038/s41598-017-00549-2 (PMC5428705; doi:10.1038/s41598-017-00549-2)

Sourdough authentication: quantitative PCR to detect the lactic acid bacterial microbiota in breads

Erica Pontonio[a], Raffaella Di Cagno[b], Jennifer Mahony[c], Alessia Lanera[a], Maria De Angelis[a], Douwe van Sinderen[c,d], and Marco Gobbetti[b*]

[a]Department of Soil, Plant and Food Sciences, University of Bari A. Moro, Bari, Italy

[b]Faculty of Science and Technology, Free University of Bolzano-Bozen, Bolzano, Italy

[c]School of Microbiology & [d]APC Microbiome Institute, University College Cork, Cork, Ireland

*Corresponding author: Marco Gobbetti, Faculty of Science and Technology, Free University of Bolzano-Bozen, Bolzano, Italy, Piazza Università, 3, 39100 Bolzano (BZ), marco.gobbetti@unibz.it

Table of contents:

- Supplementary Table S1-S7
- Supplementary Figures S1-S2

Table S1. Values of pH, Total Titratable Acidity (TTA, ml of NaOH 0.1 N), and concentration (mmol/kg) of lactic and acetic acid of breads made under pilot plant conditions (laboratory) or collected from bakeries

Bread code	pH	TTA (ml NaOH 0.1N)	Lactic acid (mmol/kg)	Acetic acid (mmol/kg)
Laboratory type I sourdough breads				
V*d*1	6.10±0.01^{ee,ff,gg,hh}	2.4±0.2^{p,q,r,s}	1.8±0.4^{c}	1.1±0.3^{e,f,g}
V*d*2	6.07±0.03^{cc,dd,ee,ff}	2.6±0.2^{s,t,u}	2.6±0.3^{d}	1.0±0.2^{c,d,e,f}
V*d*3	6.04±0.02^{aa,bb,cc,dd}	3.0±0.1^{u,v,w,x,y,z}	2.9±0.2^{d,e}	1.1±0.2^{e,f,g}
V*d*4	5.86±0.03^{pq}	3.0±0.1^{v,x,y,z}	4.8±0.3^{g}	4.0±0.4^{w,x,y,z}
V*d*5	5.89±0.02^{pq,qr,rs,st}	3.2±0.3^{x,y,z,ab,bc,de,ef}	4.7±0.2^{g}	2.9±0.3^{o,p,q,r}
V1	5.76±0.05^{op}	3.2±0.213^{x,y,z,ab,bc}	7.4±0.3^{n,o}	2.5±0.4^{m,n,o,p}
V2	5.11±0.01^{bc}	4.8±0.3^{kl,lm}	7.5±0.4^{n,o,p}	3.3±0.5^{q,r,s,t,u}
V3	4.49±0.03^{p,q}	7.2±0.1^{aa,bb,cc}	10.1±0.1^{ab,bc,cd,de,ef}	11.7±0.6^{ff}
V4	4.27±0.01^{i,j}	8.8±0.3^{ii,jj,kk,ll}	12.4±0.1^{mn,no}	9.8±0.5^{cc,dd}
V5	4.05±0.01^{b,c,d}	11.8±0.2^{ss}	23.4±0.4^{jj}	9.8±0.6^{cc,dd}
ALA*d*1	6.08±0.04^{dd,ee,ff,gg}	2.2±0.1^{o,p,q,r}	nd	nd
ALA*d*2	6.01±0.01^{xy,yz,aa,bb}	2.7±0.2^{s,t,u,v,w}	2.9±0.3^{d,e}	1.1±0.1^{e,f,g}
ALA*d*3	6.02±0.02^{yz,aa,bb,cc}	2.9±0.1^{u,v,w,x,y}	3.0±0.4^{d,e}	1.4±0.1^{e,f,g,h,i}
ALA*d*4	5.88±0.01^{pq,qr,rs}	3.4±0.3^{z,ab,bc,cd,de,ef}	3.9±0.2^{f}	2.4±0.2^{l,m,n,o}
ALA*d*5	5.65±0.02^{mn}	3.4±0.1^{z,ab,bc,cd,de,ef}	5.9±0.1^{i,j,k}	2.5±0.3^{m,n,o,p}
ALA1	5.64±0.03^{mn}	3.6±0.2^{cd,fg}	8.4±0.2^{s,t}	2.3±0.3
ALA2	4.91±0.05^{t,u,v,w}	6.0±0.1^{qr,rs}	11.5±0.1^{ij,jk}	3.2±0.4^{q,r,s,t}
ALA3	4.32±0.02^{j,k}	8.4±0.2^{gg,hh}	12.9±0.3^{op,pq}	5.4±0.2^{ef,fg,gh}
ALA4	4.17±0.03^{f,g}	10.6±0.1^{oo,pp}	18.6±0.4^{cc,dd}	7.1±0.3^{mn,no,op,pq}

Table S1. Continued

Bread code	pH	TTA<br>ml NaOH 0.1 N)	Lactic acid<br>(mmol/kg)	Acetic acid<br>(mmol/kg)
Laboratory type I sourdough breads				
ALA5	3.93±0.01^{a}	13.4±0.2^{ww}	28.8±0.1^{kk}	6.1±0.1^{ij,jk,kl}
BA*d*1	6.02±0.05^{yz,aa,bb,cc}	3.1±0.3^{v,x,y,z,ab,bc}	3.2±0.3^{e}	1.4±0.3^{e,f,g,h,i}
BA*d*2	6.01±0.02^{xy,yz,aa,bb}	3.4±0.3^{z,ab,bc,cd,de,ef}	2.8±0.4^{d,e}	1.9±0.4^{i,j,k,l}
BA*d*3	6.05±0.03^{bb,cc,dd,ee}	3.3±0.213^{y,z,ab,bc,cd,de,ef}	3.1±0.3^{d,e}	1.7±0.2^{h,i,j,k}
BA*d*4	5.69±0.01^{mn,no}	3.6±0.1^{cd,de,fg}	6.0±0.3^{j,k,l}	3.2±0.1^{q,r,s,t}
BA*d*5	5.35±0.03^{ij}	3.5±0.1^{ab,bc,cd,de,ef,fg}	5.2±0.2^{g,h}	3.5±0.3^{s,t,u,v,w}
BA1	5.47±0.02^{kl}	3.3±0.3^{y,z,ab,bc,cd,de,ef}	5.0±0.1^{g,h}	4.0±0.2^{w,x,y,z}
BA2	4.87±0.03^{t,v}	5.4±0.1^{no,op,pq}	9.3±0.3^{v,w,x,y}	9.1±0.3^{wx,xy,yz,aa}
BA3	4.34±0.01^{k,l}	8.6±0.2^{hh,ii,jj}	16.2±0.2^{uv,vw}	8.4±0.2^{rs,tu,uv}
BA4	4.18±0.01^{f,g}	10.1±0.1^{nn}	19.1±0.4^{ee}	5.9±0.4^{gh,hi,ij,jk}
BA5	4.08±0.02^{c,d}	11.1±0.2^{qq,rr}	22.5±0.3^{ii}	11.0±0.3^{ee}
CG1	4.94±0.01^{v,w,x,y}	6.2±0.2^{rs,st,tv}	9.0±0.2^{u,v}	3.0±0.3^{p,q,r,s}
CG2	4.65±0.03^{r}	7.5±0.1^{cc,dd}	9.1±0.2^{u,v,w}	6.4±0.2^{jk,kl,lm}
CG3	4.46±0.04^{o,p,q}	7.4±0.2^{bb,cc}	10.4±0.3^{de,ef,fg,gh}	9.3±0.1^{wx,xy,yz,aa,bb,cc}
CG4	4.39±0.04^{l,m}	8.4±0.2^{gg,hh}	12.3±0.1^{kl,lm,mn,no}	10.0±0.3^{dd}
CG5	4.38±0.02^{l,m}	8.7±0.2^{hh,ii,jj}	12.7±0.3^{no,op}	9.3±0.2^{wx,xy,yz,aa,bb,cc}
CG6	4.28±0.02^{i,j}	9.1±0.1^{ll}	14.9±0.4^{st,tu}	8.1±0.3^{rs,st}
CG7	4.04±0.01^{b,c}	11.7±0.3^{ss}	19.8±0.2^{ff}	10.8±0.2^{ee}
MTA1	4.92±0.03 ^{u,v,w,x}	6.8±0.1^{vw,wx,xy,yz}	11.9±0.4^{jk,kl,lm,mn}	2.9±0.2^{o,p,q,r}
MTA2	4.45±0.01^{o,p,q}	8.4±0.2^{gg,hh}	14.8±0.6^{st,tu}	4.4±0.2^{y,z,ab,bc}
MTA3	4.31±0.01^{j,k}	9.0±0.2^{kk,ll}	18.3±0.4^{bb,cc}	6.4±0.3^{jk,kl,lm}
MTA4	4.17±0.01^{f,g}	10.4±0.1^{nn,oo}	22.0±0.2^{hh}	4.5±0.3^{z,ab,bc,cd}

Table S1. Continued

Bread code	pH	TTA (ml NaOH 0.1 N)	Lactic acid (mmol/kg)	Acetic acid (mmol/kg)
		Laboratory type I sourdough breads		
MTA5	4.14±0.03^{e,f}	10.1±0.1^{nn}	19.8±0.2^{ff}	3.4±0.2^{r,s,t,u,v}
MTA6	4.10±0.01^{d,e}	11.7±0.3^{ss}	22.7±0.4^{ii}	5.0±0.4^{cd,de,ef}
MTA7	4.02±0.01^{b}	12.9±0.1^{vv}	28.8±0.2^{kk}	5.4±0.5^{ef,fg,gh}
TD1	5.22±0.05^{ef,fg,gh}	3.6±0.2^{cd,de,ef,fg}	6.5±0.2^{m}	3.1±0.4^{q,r,s,t}
TD2	4.44±0.02^{n,o,p}	7.4±0.1^{bb,cc}	11.8±0.3^{jk,kl}	5.7±0.4^{fg,gh,hi,ij}
TD3	4.38±0.01^{l,m}	8.2±0.1^{ff,gg}	13.1±0.6^{op,pq}	8.6±0.3^{tu,uv,vw}
TD4	4.34±0.03^{k,l}	8.2±0.2^{ff,gg}	13.9±0.3^{qr}	9.6±0.5^{aa,bb,cc,dd}
TD5	4.31±0.03^{j,k}	8.4±0.2^{gg,hh}	14.5±0.4^{rs,st}	5.7±0.4^{fg,gh,hi,ij}
TD6	4.28±0.01^{i,j}	8.8±0.3^{ii,jj,kk,ll}	15.9±0.4^{uv}	6.3±0.4^{jk,kl,lm}
TD7	4.24±0.01^{h,i}	9.0±0.4^{kk,ll}	16.7±0.3^{wx,yz}	7.3±0.3^{no,op,pq}
TA1	4.91±0.02^{t,u,v,w}	6.1±0.1^{rs,st}	7.6±0.4^{n,o,p,q}	4.5±0.4^{z,ab,bc,cd}
TA2	4.42±0.02^{m,n,o}	7.8±0.1^{dd,ee}	11.8±0.5^{jk,kl,lm}	5.0±0.5^{cd,de,ef}
TA3	4.31±0.01^{j,k}	8.9±0.4^{jj,kk,ll}	14.9±0.4^{st,tu}	5.5±0.3^{ef,fg,gh,hi}
TA4	4.32±0.03^{j,k}	9.0±0.3^{kk,ll}	15.1±0.4^{tu}	7.3±0.4^{no,op,pq}
TA5	4.29±0.04^{i,j,k}	9.5±0.2^{mm}	16.7±0.2^{wx,xy,yz}	5.4±0.2^{ef,fg,gh}
TA6	4.17±0.02^{f,g}	10.5±0.2^{oo,pp}	17.1±0.4^{yz,aa}	6.6±0.2^{kl,lm,mn}
TA7	4.08±0.04^{c,d}	11.2±0.1^{rr}	18.9±0.5^{dd,ee}	2.5±0.2^{m,n,o,p}
ALB1	4.94±0.02^{v,w,x,y}	6.4±0.3^{st,tu,uv}	10.3±0.4^{cd,de,ef,fg}	6.8±0.4^{lm,mn,no}
ALB2	4.61±0.04^{r}	7.4±0.1^{bb,cc}	9.3±0.3^{v,w,x,y}	8.8±0.5^{uv,vw,wx}
ALB3	4.32±0.04^{j,k}	8.5±0.2^{gg,hh,ii}	13.2±0.3^{pq}	9.7±0.5^{bb,cc,dd}
ALB4	4.21±0.05^{g,h}	10.4±0.3^{nn,oo}	16.5±0.2^{vw,wx,xy}	8.1±0.2^{rs,tu}

Table S1. Continued

**Bread code**	**pH**	**TTA (ml NaOH 0.1 N)**	**Lactic acid (mmol/kg)**	**Acetic acid (mmol/kg)**
		Laboratory type I sourdough breads		
ALB5	4.34±0.02^{k,l}	9.0±0.1^{kk,ll}	17.4±0.3^{aa}	6.3±0.6^{jk,kl,lm}
ALB6	4.17±0.02^{f,g}	12.2±0.2^{tt}	19.8±0.3^{ff}	7.5±0.6^{op,pq,qr}
ALB7	4.09±0.01^{c,d}	10.8±0.1^{pp,qq}	20.4±0.1^{gg}	9.6±0.5^{aa,bb,cc,dd}
		Laboratory baker's yeast breads		
BY1	6.11±0.01^{ff,gg,hh}	1.5±0.2^{f,g,h,i,j}	0.4±0.2	0.5±0.2^{a,b,c,d}
BY2	6.15±0.05^{hh,ii,jj}	1.4±0.1^{e,f,g,h,i}	nd	nd
BY3	6.17±0.01^{ii,jj,kk}	1.1±0.3^{b,c,d,e}	nd	nd
BY4	5.99±0.02^{vw,wx,xy}	1.7±0.1^{i,j,k,l,m,n}	0.9±0.2^{a}	0.3±0.1^{a,b}
BY5	6.13±0.05^{gg,hh,ii}	1.6±0.2^{g,i,j}	nd	nd
BY6	6.15±0.02^{hh,ii,jj}	1.6±0.2^{g,i,j}	nd	nd
BY7	6.25±0.01^{ll,nn,oo}	0.8±0.1^{a,b}	nd	nd
BY8	6.23±0.04^{ll}	0.6±0.1^{a}	nd	nd
BY9	6.25±0.02^{ll,oo}	0.9±0.1^{a,b,c}	nd	nd
BY10	6.14±0.03^{hh,ii}	1.1±0.1^{b,c,d,e}	0.1±0.1^{a}	0.5±0.1^{a,b,c,d}
BY11	6.17±0.01^{ii,jj,kk}	1.3±0.2^{d,e,f,g,h}	nd	nd
BY12	6.21±0.02^{kk,ll}	1.2±0.1^{c,d,e,f}	nd	nd
		Dried laboratory sourdough breads		
P1	5.27±0.06^{gh,hi}	4.8±0.3^{kl,lm}	5.1±0.4^{g,h}	3.3±0.1^{q,r,s,t,u}
P2	5.22±0.02^{ef,fg,gh}	5.2±0.3l^{m,mn,no}	4.7±0.2^{g}	2.9±0.3^{o,p,q,r,s}
P3	5.07±0.03^{z,ab,bc}	6.1±0.2^{rs,st}	7.6±0.3^{n,o,p,q}	1.0±0.2^{c,d,e,f,g}
P4	5.01±0.01^{z,ab}	6.2±0.2^{rs,st,tu}	9.0±0.2^{u,v}	2.2±0.2^{k,l,m}

Table S1. Continued

Bread code	pH	TTA (ml NaOH 0.1 N)	Lactic acid (mmol/kg)	Acetic acid (mmol/kg)
		Dried laboratory sourdough breads		
P5	4.99±0.04^{y,z,ab}	7.0±0.3^{xy,yz,aa}	10.2±0.4^{bc,cd,de,ef}	3.2±0.4^{q,r,s,t}
P6	4.87±0.02^{t,u}	7.4±0.1^{bb,cc}	16.2±0.6^{uv,vw}	2.6±0.1^{m,n,o,p}
P7	5.31±0.02^{hi,ij}	3.6±0.1^{cd,de,ef,fg}	6.7±0.2^{m}	1.1±0.2^{e,f,g}
P8	5.25±0.02^{fg,gh,hi}	4.1±0.1^{gh,hi,ij}	7.6±0.4^{n,o,p,q}	2.4±0.2^{l,m,n,o}
P9	5.12±0.02^{bc}	5.5±0.1^{op,pq}	8.4±0.4^{s,t}	2.0±0.6^{j,k,l,m}
P10	5.48±0.02^{kl}	3.8±0.1^{fg,gh}	5.2±0.1^{g,h}	1.2±0.3^{e,f,g,h}
P11	5.39±0.01^{jk}	3.9±0.1^{fg,gh,ij}	5.5±0.1^{h,i}	1.2±0.1^{e,f,g,h}
P12	5.24±0.02^{fg,gh}	4.7±0.1^{jk,kl,lm}	7.9±0.2^{o,p,q,r,s}	1.9±0.4^{i,j,k,l}
P13	5.49±0.02 ^{kl}	3.8±0.1^{fg,gh}	6.8±0.2^{m}	1.1±0.2^{e,f,g}
P14	5.49±0.02 ^{kl}	3.8±0.1^{fg,gh}	6.9±0.2^{m}	1.1±0.2^{e,f,g}
P15	5.36±0.02 ^{ij}	4.2±0.2^{hi,ij}	7.5±0.2^{n,o,p}	1.3±0.3^{e,f,g,h}
P16	5.51±0.01^{kl}	3.9±0.1^{fg,gh,hi}	7.5±0.1^{n,o,p}	1.2±0.2^{e,f,g,h}
P17	5.50±0.02^{kl}	4.1±0.1^{gh,hi,ij}	7.8±0.4^{o,p,q,r,s}	1.2±0.2^{e,f,g,h}
P18	5.32±0.02^{ij}	4.4±0.1^{ij,jk}	9.1±0.5^{u,v,w}	1.6±0.4^{g,h,i,j}
P19	5.05±0.01^{ab,bc}	6.1±0.2^{rs,st}	10.0±0.2^{ab,bc,cd,de}	1.2±0.2^{e,f,g,h}
P20	5.02±0.02^{z,ab}	5.9±0.1^{qr,rs}	10.1±0.5^{ab,bc,cd,de}	1.1±0.4^{e,f,g}
P21	4.86±0.01^{t}	6.2±0.3^{rs,st,tu}	12.9±0.3^{op,pq}	2.1±0.1^{k,l,m}
		Bakery sourdough breads		
A	5.01±0.01^{z,ab}	6.2±0.2^{rs,st,tu}	9.0±0.2^{u,v}	2.2±0.2^{k,l,m}
B	4.86±0.01^{t}	7.0±0.1^{xy,yz,aa}	12.3±0.3^{kl,lm,mn,no}	9.5±0.4^{yz,aa,bb,cc,dd}
C	5.12±0.03^{bc}	5.6±0.2^{op,pq}	7.3±0.3^{n}	6.4±0.6^{jk,kl,lm}

Table S1. Continued

Bread code	pH	TTA (ml NaOH 0.1 N)	Lactic acid (mmol/kg)	Acetic acid (mmol/kg)
		Bakery sourdough breads		
D	5.11±0.04^{bc}	6.6±0.3^{uv,vw,wx}	8.1±0.5^{q,r,s}	1.0±0.1^{d,e,f}
E	5.32±0.02^{ij}	3.5±0.2 ^{ab,cd,de,ef,fg}	6.6±0.5^{m}	3.5±0.3^{s,t,u,v,w}
I	5.21±0.05^{ef,fg,gh}	6.1±0.2^{rs,st}	10.6±0.3^{ef,fg,gh}	4.2±0.3^{x,y,z,ab}
L	5.02±0.02^{z}	6.5±0.1^{tu,uv,vw}	9.4±0.4^{v,w,x,y,z}	5.0±0.6^{cd,de,ef}
M	5.24±0.02^{fg,gh}	4.4±0.1^{ij,jk}	9.1±0.5^{u,v,w}	7.9±0.4^{qr,rs,st}
N	4.76±0.04^{n}	7.0±0.3^{xy,yz,aa}	10.2±0.4^{bc,cd,de,ef}	7.2±0.4^{no,op,pq}
O	4.96±0.02^{x,y,ab}	7.4±0.1^{bb,cc}	16.2±0.6^{uv,vw}	10.6±0.1^{ee}
P	5.01±0.01^{z}	7.8±0.2^{dd,ee}	12.1±0.6^{kl,lm,mn}	9.2±0.5^{wx,xy,yz,aa,bb}
Q	5.71±0.02^{no,op}	3.0±0.1^{u,v,w,x,y,z}	3.1±0.2^{d,e}	0.9±0.1^{c,d,e}
T	4.39±0.03^{l,m,n}	7.2±0.3^{aa,bb,cc}	14.2±0.4^{qr,rs}	7.0±0.5^{mn,no,op}
U	5.01±0.03^{z,ab}	6.5±0.2^{tu,uv,vw}	9.7±0.2^{x,y,z,ab,bc}	5.1±0.3^{de,ef}
V	4.87±0.05^{t,v}	6.6±0.2^{uv,vw,wx}	12.3±0.3^{lm,mn,no}	9.7±0.3^{bb,cc,dd}
Z	5.04±0.01^{z}	6.7±0.1^{uv,vw,wx,xy}	9.8±0.4^{y,z,ab,bc,cd}	6.3±0.4^{jk,kl,lm}
A2	5.01±0.02^{z,ab}	6.6±0.3^{uv,vw,wx}	10.1±0.2^{ab,bc,cd,de,ef}	6.2±0.4^{ij,jk,kl}
A3	5.22±0.04^{ef,fg,gh}	5.6±0.2^{op,pq}	8.1±0.6^{q,r,s}	4.7±0.5^{ab,bc,cd,de}
A4	4.61±0.02^{r}	7.8±0.2^{dd,ee}	10.1±0.2^{ab,bc,cd,de,ef}	7.6±0.3^{pq,qr}
A5	4.91±0.02^{t,u,v,w}	6.9±0.2^{wx,xy,yz,aa}	11.4±0.2^{ij,jk}	9.4±0.3^{xy,yz,aa,bb,cc}
A6	4.86±0.05^{t}	7.0±0.1^{xy,yz,aa}	8.2±0.5^{r,s}	5.9±0.3^{gh,hi,ij,jk}
A7	4.90±0.01^{t,u,v,w}	7.1±0.1^{yz,aa,bb}	10.8±0.3^{fg,gh,hi}	11.5±0.3^{ff}
A8	4.95±0.04^{w,x,y}	8.0±0.2^{ee,ff}	10.4±0.4^{de,ef,fg,gh}	6.3±0.2^{jk,kl,lm}
A9	5.41±0.02^{jk}	4.1±0.1^{gh,hi,ij}	7.2±0.4^{n}	3.4±0.2^{r,s,t,u,v}

Table S1. Continued

Bread code	pH	TTA (ml NaOH 0.1 N)	Lactic acid (mmol/kg)	Acetic acid (mmol/kg)
		Bakery sourdough breads		
A11	5.11±0.02^{bc}	5.7±0.2^{pq,qr}	9.2±0.5^{u,v,w,x}	2.3±0.3^{l,m,n}
A16	5.65±0.02^{mn}	4.0±0.1^{gh,hi}	7.5±0.3^{n,o,p}	3.8±0.4^{u,v,w,x}
A17	5.11±0.01^{bc}	5.5±0.1^{op,pq}	8.7±0.5^{t,u}	4.8±0.4^{bc,cd,de}
A18	5.01±0.06^{z,ab}	5.0±0.3^{lm,mn}	5.1±0.4^{g,h}	3.3±0.1^{q,r,s,t,u}
A19	5.49±0.05^{kl}	4.3±0.3^{hi,ij,jk}	10.8±0.3^{fg,gh,hi}	4.8±0.3^{bc,cd,de}
A21	5.47±0.02^{kl}	4.6±0.4^{jk,kl}	7.7±0.1^{n,o,p,q,r}	2.8±0.2^{n,o,p,q}
A24	5.57±0.05^{lm}	3.2±0.1^{x,y,z,ab,bc,cd,de,ef}	5.1±0.2^{g,h}	2.3±0.2^{l,m,n}
A26	5.47±0.05^{kl}	5.0±0.2^{lm,mn}	9.1±0.3^{u,v,w}	4.2±0.4^{x,y,z,ab}
A29	5.24±0.04^{fg,gh}	4.9±0.1^{kl,lm}	7.2±0.5^{n}	1.4±0.2^{e,f,g,h,i}
A31	5.01±0.01^{z,ab}	6.5±0.1^{tu,uv,vw}	10.9±0.2^{gh,hi}	4.0±0.1^{w,x,y,z}
A32	5.34±0.06^{ij}	5.1±0.5^{lm,mn,no}	7.9±0.4^{o,p,q,r}	2.3±0.3^{l,m,n}
A37	5.15±0.02^{bc,cd}	5.0±0.1^{lm,mn}	6.2±0.3^{k,l,m}	3.5±0.4^{s,t,u,v,w}
A40	4.20±0.04^{g,h}	9.0±0.2^{kk,ll}	18.0±0.4^{bb}	7.5±0.3^{op,pq,qr}
A41	5.15±0.03^{bc,cd,de}	5.6±0.1^{op,pq}	9.2±0.3^{u,v,w,x}	3.6±0.2^{t,u,v,w}
A42	3.94±0.01^{a}	12.7±0.4^{vv}	30.4±0.3^{ii}	9.0±0.3^{vw,wx,xy,yz}
A43	5.75±0.01^{op}	3.0±0.1^{u,v,w,x,y,z}	7.2±0.1^{n}	0.5±0.2^{a,b,c}
A44	5.18±0.04^{cd,de,ef}	5.0±0.2^{lm,mn}	9.6±0.2^{w,x,y,z,ab}	4.8±0.2^{bc,cd,de}
A47	4.50±0.02^{q}	7.8±0.3^{dd,ee}	11.2±0.4^{hi,ij}	6.8±0.4^{lm,mn,no}
A51	5.30±0.01^{hi,ij}	4.0±0.3^{gh,hi}	5.7±0.4^{i,j}	3.9±0.2^{v,w,x,y}
A52	5.20±0.01^{de,ef,fg}	4.2±0.1^{hi,ij}	5.1±0.3^{g,h}	3.2±0.3^{q,r,s,t}
2	4.80±0.02^{n}	5.4±0.3^{no,op,pq}	9.0±0.2^{u,v}	9.2±0.2^{wx,xy,yz,aa,bb}

Table S1. Continued

Bread code	pH	TTA (ml NaOH 0.1 N)	Lactic acid (mmol/kg)	Acetic acid (mmol/kg)
		Bakery sourdough breads		
6	5.10±0.04^{bc}	5.4±0.1^{no,op,pq}	7.6±0.2^{n,o,p,q}	4.0±0.1^{w,x,y,z}
8	4.94±0.03^{v,w,x,y}	6.7±0.2^{uv,vw,wx,xy}	10.3±0.3^{cd,de,ef,fg}	6.0±0.3^{hi,ij,jk}
11	5.75±0.01^{op}	2.9±0.1^{u,v,w,x,y}	5.5±0.1^{h,i}	1.2±0.3^{e,f,g,h}
12	5.12±0.02^{bc}	5.1±0.2^{lm,mn,no}	7.3±0.2^{n}	5.5±0.4^{ef,fg,gh,hi}
13	5.14±0.04^{bc,cd}	5.3±0.3^{mn,no,op}	8.0±0.1^{p,q,r,s}	5.2±0.3^{de,ef,fg}
14	4.89±0.04^{t,v,w}	5.4±0.2^{no,op,pq}	9.9±0.3^{z,ab,bc,cd,de}	5.7±0.4^{fg,gh,hi,ij}
15	5.21±0.02^{ef,fg,gh}	4.4±0.2^{ij,jk}	6.7±0.1^{m}	3.9±0.4^{v,w,x,y}
26	5.26±0.05^{gh,hi}	4.2±0.3^{hi,ij}	6.4±0.3^{l,m}	4.2±0.4^{x,y,z,ab}
27	4.99±0.04^{y,z,ab}	6.8±0.1^{vw,wx,xy,yz}	8.3±0.4^{s,t}	1.6±0.4^{g,h,i,j}
28	5.04±0.02^{z}	6.1±0.2^{rs,st}	9.5±0.2^{v,w,x,y,z}	1.3±0.3^{e,f,g,h}
29	5.01±0.01^{z,ab}	6.2±0.3^{rs,st,tu}	7.9±0.3^{o,p,q,r,s}	2.1±0.1^{j,k,l,m}
32	4.89±0.01^{t,v,w}	6.0±0.3^{rs}	10.3±0.2^{cd,de,ef,fg}	8.9±0.3^{uv,vw,wx,xy}
		Bakery baker's yeast breads		
R	6.00±0.05^{wx,xy,yz}	3.0±0.1^{u,v,w,x,y,z}	nd	nd
1	6.30±0.03^{mm,nn,oo}	2.0±0.2^{k,l,m}	1.1±0.1^{a}	1.6±0.3^{g,h,i,j}
4	6.30±0.02^{mm,nn}	1.6±0.3^{g,i,j}	1.7±0.2^{b,c}	1.6±0.3^{g,h,i,j}
5	6.20±0.04^{jj,kk,ll}	2.0±0.2^{k,o}	nd	nd
9	6.20±0.04^{jj,kk,ll}	2.0±0.2^{k,l,o}	1.2±0.4^{a}	0.5±0.4^{b,c,d}
10	6.00±0.04^{wx,xy,yz,}	2.4±0.1^{p,s}	1.3±0.2^{a,b}	1.2±0.4^{e,f,g,h}
16	5.90±0.01^{pq,qr,rs,st}	3.1±0.2^{v,x,y,z,ab,bc}	1.2±0.4^{a}	1.5±0.3^{f,g,h,i}
18	6.20±0.02^{jj,kk,ll}	2.2±0.3^{o,p,q,r}	nd	nd

Table S1. Continued

**Bread code**	**pH**	**TTA (ml NaOH 0.1 N)**	**Lactic acid (mmol/kg)**	**Acetic acid (mmol/kg)**
		Bakery baker's yeast breads		
19	6.40 ± 0.02^{pp}	$2.8\pm0.1^{t,u,v,w,x}$	nd	nd
20	$6.00\pm0.02^{wx,xy,yz,aa,bb}$	$2.0\pm0.3^{k,o}$	nd	nd
22	$6.20\pm0.03^{jj,kk,ll}$	$1.8\pm0.2^{j,k,l,m,n}$	nd	nd
23	6.30 ± 0.02^{mm}	$1.4\pm0.3^{e,f,g,h,i}$	nd	nd
30	$6.10\pm0.03^{ee,ff,gg,hh}$	$2.0\pm0.1^{k,o}$	1.1 ± 0.2^{a}	$1.5\pm0.4^{f,g,h,i}$
31	$6.00\pm0.02^{wx,xy,yz,aa,bb}$	$1.6\pm0.3^{g,i,j}$	nd	nd
A10	$6.05\pm0.01^{bb,cc,dd,ee}$	$2.5\pm0.1^{p,s,t}$	nd	nd
A12	5.85 ± 0.01^{pq}	$3.1\pm0.2^{v,x,y,z,ab,bc}$	nd	nd
A13	$5.89\pm0.01^{pq,qr,rs,st}$	$3.0\pm0.2^{v,x,y,z}$	nd	nd
A14	5.85 ± 0.04^{pq}	$3.0\pm0.1^{v,x,y,z}$	nd	nd
A15	$5.99\pm0.04^{vw,wx,xy,yz,aa}$	$3.0\pm0.3^{v,x,y,z}$	nd	nd
A20	$6.01\pm0.04^{xy,yz,aa,bb}$	$3.0\pm0.1^{v,w,x,y,z}$	nd	nd
A22	$6.03\pm0.05^{aa,bb,cc,dd}$	$2.0\pm0.1^{k,o}$	nd	nd
A23	$5.97\pm0.06^{uv,vw,wx,xy,yz}$	$3.1\pm0.3^{v,x,y,z,ab,bc}$	nd	nd
A25	$5.92\pm0.02^{qr,rs,st,tu,uv}$	$3.0\pm0.2^{u,v,w,x,y,z}$	nd	nd
A27	$6.12\pm0.02^{ff,gg,hh,ii}$	$3.0\pm0.2^{u,v,w,x,y,z}$	nd	nd
A28	$6.00\pm0.04^{wx,xy,yz,aa,bb}$	$2.4\pm0.1^{p,q,s}$	nd	nd
A30	$6.12\pm0.02^{ff,gg,hh,ii}$	$3.8\pm0.2^{fg,gh}$	nd	nd
A33	$5.93\pm0.04^{rs,st,tu,uv}$	$3.0\pm0.3^{u,v,w,x,y,z}$	nd	nd
A34	$5.95\pm0.04^{tu,uv,vw,wx}$	$2.8\pm0.1^{t,u,v,w,x}$	nd	nd
A35	$6.05\pm0.03^{bb,cc,dd,ee}$	$1.6\pm0.1^{g,i,j}$	nd	nd

Table S1. Continued

Bread code	pH	TTA (ml NaOH 0.1 N)	Lactic acid (mmol/kg)	Acetic acid (mmol/kg)
		Bakery baker's yeast breads		
A48	6.10±0.03[ee,ff,gg,hh]	1.2±0.2[c,d,e,f]	nd	nd
A49	5.87±0.05[pq,qr]	3.2±0.13[x,y,z,ab,bc,ef]	nd	nd
A50	6.10±0.02[ee,ff,gg,hh]	1.0±0.2[b,c,d]	nd	nd
A45	5.96±0.02[uv,vw,wx,xy]	2.2±0.1[o,p,q,r]	nd	nd
A46	5.95±0.04[uv,vw,wx]	2.6±0.3[s,t,u,w]	nd	nd
A39	5.90±0.02[pq,qr,rs,st,tu]	1.4±0.2[e,f,g,h,i]	nd	nd
A38	5.94±0.02 [st,tu,uv,vw]	2.0±0.3[k,l,m,n,o]	nd	nd

nd, not detectable

The ingredients and technology parameters used for bread making under pilot plant conditions (laboratory) are reported in Table 2. Description of commercial breads collected from bakeries are reported in Supplementary Table S6.

Values are means ± standard deviation of three batches analysed in triplicate (n = 9). Values within a column with different superscript single and/or pairs letters are significantly different (P<0.05).

Table S2. Sourdough lactic acid bacteria and flour contaminant strains used in the study

**Strain**[a]	**Culture Collection***	**Strain**[a]	**Culture Collection***
	**Lactic acid bacteria**		
L. fermentum<br>ATCC 14931	ATCC	*L. plantarum*<br>4-4, 3-3, 1M, 5-5, 3DM, WCFS1, DC400	DISSPA-MICRO
L. fermentum<br>F1	DISSPA-MICRO	*L. delbrueckii*<br>B15Z	DISSPA-MICRO
W. confusa<br>DSM 20196	DSM	*L. rossiae*<br>DSM 18514	DSMZ
P. pentosaceus<br>PP10, PP5	DISSPA-MICRO	*L. rossiae*<br>LB5, 8-5	DISSPA-MICRO
L. curvatus<br>ATCC 25601	ATCC	*L. sanfranciscensis*<br>7A, 13, A1, E14, E21, 274	DISSPA-MICRO
Leuc. citreum<br>22A	DISSPA-MICRO	*L. brevis*<br>LB4	DISSPA-MICRO
W. cibaria<br>5S, 7S	DISSPA-MICRO	*L. hilgardii*<br>ATCC 8290	ATCC
L. pentosus<br>ATCC 8041	ATCC	*L. hammesii*<br>BCCM/LMG 23074	BCCM
L. amylovorus<br>L_amy	DISSPA-MICRO	*E. faecium*<br>22	ISPA-CNR
L. amylovorus<br>ATCC 33620	ATCC	*E. faecium*<br>DSM 20477	DSMZ

Table S2. Continued

**Strain**[a]	**Culture Collection***	**Strain**[a]	**Culture Collection***
	**Lactic acid bacteria**		
Lc. lactis 10γ	DISSPA-MICRO	*E. faecalis* ATCC 23655	ATCC
L. acidifarinae DSM 19394	DSMZ	*Enterococcus faecalis* 40	ISPA-CNR
L. frumenti DSM 13145	DSMZ	*L. farciminis* DSM 20184	DSMZ
L. namurensis DSM 19117	DSMZ	*L. alimentarius* ATCC 29643	ATCC
L. nantensis DSM 16982	DSMZ	*L. casei* ATCC-393	ATCC
L. panis DSM 6035	DSMZ	*L. paralimentarius* DSM 13238	DSMZ
L. pontis DSM 8475	DSMZ	*P. acidilactici* DSM 20238	DSMZ
L. reuteri ATCC-23272	ATCC	*L. acidophilus* ATCC-4356	ATCC
L. helveticus ATCC 15009	ATCC	*L. amylolyticus* DSM 11664	DSMZ
Leuc. mesenteroides subsp. *cremoris* DSM 20346	DSMZ	*L. crispatus* DSM 20584	DSMZ

Table S2. Continued

Strain[a]	Culture Collection*	Strain[a]	Culture Collection*
**Lactic acid bacteria**			
Leuc. mesenteroides subsp. *dextranicum* DSM 46216	DSMZ	*Leuc. mesenteroides* subsp. *mesenteroides* DSM 20343	DSMZ
L. kunkeei LKA, LKB	DISSPA-MICRO	*Leuc. mesenteroides* subsp. *mesenteroides* LM5	DISSPA-MICRO
**Flour contaminants**			
St. capitis Sc1	DISSPA-MICRO	*Atl. hermannii* Eh1	DISSPA-MICRO
Se. marcescens Sm1	DISSPA-MICRO	*Pa. agglomerans* Pag 37	DISSPA-MICRO
B. megaterium Bm1	DISSPA-MICRO	*Acinetobacter* sp. A1	DISSPA-MICRO
En. aurogenes Ea1, Ea2, Ea3	ISPA-CNR	*Enterobacter* sp. E1	DISSPA-CHEM
Ac. calcoaceticus Ac1	ISPA-CNR	*Pantoea* sp. Pan 38	DISSPA-MICRO
Pa. agglomerans Pa3	ISPA-CNR	*Bacillus* sp. B2	DiSSPA-MICRO
Ps. fluorescent Pf1	ISPA-CNR	*Pseudomonas* sp. P1	DISSPA-CHEM

Table S2. Continued

**Strain**[a]	**Culture Collection***	**Strain**[a]	**Culture Collection***
	**Flour contaminants**		
Rhizobium sp. R1	DISSPA-CHEM	*Commamonas* sp. C1	DISSPA-CHEM
Delftia sp. D1	DISSPA-CHEM	*Staphylococcus* sp. S1	DISSPA-CHEM
Staphylococcus sp. Sta 41	DISSPA-MICRO	*Erwinia* sp. Er1	DISSPA-MICRO
Sphingomonas sp. Sp1	DISSPA-MICRO		

* BCCM/LMG, Belgian Coordinated Collections of Microorganisms; DiSSPA-MICRO, Section of Food Microbiology of the Department of Soil, Plant and Food Sciences, University of Bari A. Moro, Italy; DISSPA-CHEM, Section of Agricultural Chemistry of the Department of Soil, Plant and Food Sciences, University of Bari A. Moro, Italy; ATCC, American Type Culture Collection; DSMZ, Deutsche Sammlung von Mikroorganismen and Zellkulturen; ISPA-CNR, Institute of Sciences of Food Production - National Research Council of Italy (CNR), Bari, personal Collection of Dr. P. Lavermicocca.

[a] *Ac., Acinetobacter; Atl., Atlantibacter*; *B., Bacillus*; *E., Enterococcus*; *En., Enterobacter*; *L., Lactobacillus*; *Lc., Lactococcus*; *Leuc., Leuconostoc*; *W., Weiessella*; *Pa., Pantoea*; *Ps., Pseudomonas*; *Se., Serratia*; *St., Staphylococcus*.

Table S3. Cell density (Log cfu/g), cycle threshold (C_T), and gene copy number (Log gene copy/g) of breads inoculated with pure culture of *Lactobacillus plantarum* WCFS1 used to generate the calibration curve

**Cell density (Log cfu/g)**	**Cycle threshold (C_T)**	**Gene copies number (Log gene copy/g)**
5.8±0.1^{a}	28.9±0.2	6.5±0.2^{a}
6.5±0.2^{b}	27.7±0.1	7.2±0.1^{b}
6.8±0.1^{b,c}	27.0±0.2	7.5±0.2^{b,c}
7.1±0.2^{c}	26.1±0.1	7.8±0.1^{c}
7.8±0.3^{d}	24.6±0.1	8.5±0.1^{d}
8.1±0.1^{d}	24.1±0.3	8.8±0.3^{d}
8.8±0.1^{e}	22.7±0.2	9.5±0.2^{e}
9.1±0.2^{e}	21.4±0.2	9.8±0.2^{e}
9.8±0.3^{f}	19.8±0.3	10.5±0.3^{f}

Values are means ± standard deviation of three batches analysed in triplicate (n = 9). Values within a column with different superscript letters are significantly different ($P<0.05$).

Table S4. Cycle threshold (C_T) and gene copy number (Log gene copy/g) of type I sourdough, baker's yeast and dried sourdough breads made under pilot plant conditions (laboratory) used to define the limit of detection of the qPCR protocol

**Bread code**	**Cycle threshold (C_T)**	**Gene copy number (Log gene copy/g)**
	Type I sourdough breads	
V*d*1	27.3±0.2	$7.3±0.2^{f,g,h}$
V*d*2	26.6±0.5	$7.6±0.5^{f,h,i,j,k}$
V*d*3	26.2±0.3	$7.8±0.3^{f,h,i,j,k,l}$
V*d*4	25.9±0.3	$7.9±0.3^{f,h,i,j,k,l,m}$
V*d*5	26.8±0.2	$7.5±0.2^{f,g,h,i}$
V1	25.8±0.2	$8.0±0.2^{f,h,i,j,k,l,m,n}$
V2	23.0±0.3	$9.2±0.3^{p,q,r,s,t,u,v}$
V3	22.2±0.2	$9.5±0.2^{s,t,u,v}$
V4	21.4±0.3	$9.9±0.3^{v}$
V5	21.7±0.2	$9.7±0.2^{u,v}$
ALA*d*1	26.8±0.4	$7.5±0.4^{f,h,i,k}$
ALA*d*2	24.9±0.3	$8.3±0.3^{i,j,k,l,m,n,o,p,q}$
ALA*d*3	24.5±0.2	$8.5±0.2^{i,l,m,n,o,p,q,r,s}$
ALA*d*4	23.9±0.9	$8.8±0.9^{m,n,o,p,q,r,s,t,u}$
ALA*d*5	23.6±0.4	$8.9±0.4^{m,n,o,p,q,r,s,t,u,v}$
ALA1	22.4±0.5	$9.5±0.5^{s,t,u,v}$
ALA2	22.8±0.4	$9.3±0.4^{q,r,s,t,u,v}$
ALA3	22.7±0.4	$9.3±0.4^{q,r,s,t,u,v}$
ALA4	21.8±0.6	$9.7±0.6^{u,v}$

Table S4. Continued

Bread code	Cycle threshold (C_T)	Gene copy number (Log gene copy/g)
	Type I sourdough breads	
ALA5	21.9±0.3	9.6±0.3^{t,u,v}
BA*d*1	27.4±0.3	7.2±0.3^{f,g}
BA*d*2	25.1±0.3	8.3±0.3^{i,j,k,l,m,n,o,p,q}
BA*d*3	24.6±0.3	8.5±0.3^{i,k,l,m,n,o,p,q,r,s}
BA*d*4	24.0±0.3	8.7±0.3^{m,n,o,p,q,r,s,t,u}
BA*d*5	23.4±0.4	9.0±0.4^{n,o,p,q,r,s,t,u,v}
BA1	23.2±0.8	9.1±0.8^{o,p,q,r,s,t,u,v}
BA2	23.2±0.4	9.1±0.4^{o,p,q,r,s,t,u,v}
BA3	23.2±0.2	9.1±0.2^{o,p,q,r,s,t,u,v}
BA4	22.3±0.3	9.5±0.3^{s,t,u,v}
BA5	22.0±0.3	9.6±0.3^{t,u,v}
CG1	26.1±0.3	7.8±0.3^{f,h,i,j,k,l}
CG2	24.0±0.5	8.7±0.5^{m,n,o,p,q,r,s,t,u}
CG3	24.3±0.6	8.6±0.6^{m,n,o,p,q,r,s,t}
CG4	22.6±0.1	9.3±0.1^{q,r,s,t,u,v}
CG5	23.4±0.9	9.0±0.9^{n,o,p,q,r,s,t,u,v}
CG6	23.4±0.2	9.0±0.2^{n,o,p,q,r,s,t,u,v}
CG7	22.9±0.4	9.2±0.4^{p,q,r,s,t,u,v}
MTA1	26.2±0.3	7.8±0.3^{f,h,i,j,k,l}
MTA2	25.3±0.9	8.2±0.9^{h,i,j,k,l,m,n,o,p}
MTA3	25.5±0.1	8.1±0.1^{f,h,i,j,k,l,m,n,o}

Table S4. Continued

**Bread code**	**Cycle threshold (C_T)**	**Gene copy number (Log gene copy/g)**
	Type I sourdough breads	
MTA4	24.6±0.7	8.5±0.7$^{i,k,l,m,n,o,p,q,r,s\ i}$
MTA5	25.8±0.1	8.0±0.1^{f,h,i,j,k,l,m,n}
MTA6	25.8±0.4	8.0±0.4^{f,h,i,j,k,l,m,n}
MTA7	25.8±0.3	8.0±0.3^{f,h,i,j,k,l,m,n}
TD1	24.3±1.0	8.6±1.0^{m,n,o,p,q,r,s,t}
TD2	23.9±0.4	8.8±0.4^{m,n,o,p,q,r,s,t,u}
TD3	24.3±0.5	8.6±0.5^{m,n,o,p,q,r,s,t}
TD4	23.1±0.1	9.1±0.1^{o,p,q,r,s,t,u,v}
TD5	24.6±0.4	8.5±0.4^{i,l,m,n,o,p,q,r,s}
TD6	23.5±0.6	9.0±0.6^{n,o,p,q,r,s,t,u,v}
TD7	23.1±1.5	9.1±1.5^{o,p,q,r,s,t,u,v}
TA1	22.7±1.3	9.3±1.3^{q,r,s,t,u,v}
TA2	21.9±0.6	9.7±0.6^{u,v}
TA3	22.0±0.8	9.6±0.8^{w}
TA4	23.9±0.6	8.8±0.6^{m,n,o,p,q,r,s,t,u}
TA5	24.8±0.5	8.4±0.5^{i,j,k,l,m,n,o,p,q}
TA6	24.8±0.9	8.4±0.9^{i,j,k,l,m,n,o,p,q,r}
TA7	23.6±0.0	8.9±0.0^{m,n,o,p,q,r,s,t,u,v}
ALB1	23.3±0.6	9.0±0.6^{n,o,p,q,r,s,t,u,v}
ALB2	24.6±1.5	8.5±1.5^{i,k,l,m,n,o,p,q,r,s}
ALB3	23.4±0.4	9.0±0.4^{n,o,p,q,r,s,t,u,v}

Table S4. Continued

**Bread code**	**Cycle threshold (C_T)**	**Gene copy number (Log gene copy/g)**
	Type 1 sourdough breads	
ALB4	22.7±0.1	9.3±0.1^{q,r,s,t,u,v}
ALB5	24.2±0.6	8.6±0.6^{m,n,o,p,q,r,s,t}
ALB6	24.0±0.5	8.7±0.5^{m,n,o,p,q,r,s,t,u}
ALB7	22.5±0.1	9.4±0.1^{r,s,t,u,v}
	Baker’s yeast breads	
BY1	29.6±0.2	6.3±0.2^{c,d,e}
BY2	30.4±0.1	5.9±0.1^{b,c,d}
BY3	29.8±0.4	6.2±0.4^{c,d,e}
BY4	30.1±0.1	6.1±0.1^{a,b,c}
BY5	29.5±0.5	6.3±0.4^{a,b,c}
BY6	30.2±0.4	6.0±0.4^{a,b,c}
BY7	29.9±0.1	6.2±0.1^{a,b,c}
BY8	31.7±0.6	5.4±0.6^{a}
BY9	30.3±0.3	6.0±0.3^{a,b,c}
BY10	31.3±0.6	5.5±0.6^{a,b}
BY11	31.3±0.4	5.5±0.4^{a,b}
BY12	29.7±0.2	6.2±0.2^{a,b,c}
	Dried laboratory sourdough breads	
P1	30.0 ± 0.1	6.1± 0.1^{b,c}
P2	26.8 ± 0.2	7.5± 0.2^{f,g,h,i}
P3	26.7 ± 0.1	7.6± 0.1^{f,h,i}

Table S4. Continued

Bread code	Cycle threshold (C_T)	Gene copy number (Log gene copy/g)
	Dried laboratory sourdough breads	
P4	26.1 ± 0.2	7.8± 0.2^{f,h,i}
P5	23.6 ± 0.1	8.9± 0.1^{m,n,o,p,q,r,s,t,u}
P6	21.2 ± 0.2	10.0± 0.2^{v}
P7	27.6 ± 0.1	7.2± 0.1^{f,g}
P8	24.7 ± 0.1	8.4± 0.1^{i,j,k,l,m,n,o,p,q,r}
P9	21.9 ± 0.3	9.6± 0.3^{t,u,v}
P10	31.4 ± 0.1	5.5± 0.1^{a}
P11	27.8 ± 0.1	7.1± 0.1^{f,g}
P12	22.7 ± 0.2	9.3± 0.2^{q,r,s,t,u,v}
P13	30.9 ± 0.3	5.7± 0.3^{a,b}
P14	27.2 ± 0.1	7.3± 0.1^{f,g,h}
P15	26.7 ± 0.1	7.5± 0.1^{f,g,h,i}
P16	28.3 ± 0.1	6.8± 0.1^{b,c}
P17	25.1 ± 0.3	8.3± 0.3^{i,j,k,l,m,n,o,p,q}
P18	23.3 ± 0.2	9.1± 0.2^{o,p,q,r,s,t,u,v}
P19	25.7± 0.1	8.0± 0.1^{f,h,i,m,n}
P20	23.2 ± 0.1	9.1± 0.1^{o,p,q,r,s,t,u,v}
P21	21.5 ± 0.5	9.8± 0.5^{t,u,v}

The ingredients and technology parameters used for bread making under pilot plant conditions (laboratory) are reported in Table 2.

Values are means ± standard deviation of three batches analysed in triplicate (n = 9). Values within a column with different superscript letters are significantly different (P<0.05).

Table S5. Cycle threshold (C_T) and gene copy number (Log gene copy/g) of sourdough and baker's yeast breads collected from bakeries

**Bread code**	**Cycle threshold (C_T)**	**Gene copy number (Log gene copy/g)**
	Sourdough breads	
A	26.0±0.3	7.9±0.3^{m,n,o,p,q}
B	22.1±0.7	9.6±0.7^{s,t,u,v,w,x}
C	22.1±0.5	9.6±0.5^{s,t,u,v,w,x}
D	22.2±0.9	9.5±0.9^{r,s,t,u,v,w,x}
E	22.1±0.2	9.6±0.2^{s,t,u,v,w,x}
I	22.7±0.4	9.3±0.4^{q,r,s,t,u,v,w,x}
L	21.7±0.3	9.7±0.3^{t,u,v,w,x}
M	22.6±0.2	9.3±0.2^{q,r,s,t,u,v,w,x}
N	23.6±0.4	8.9±0.4^{n,o,p,q,r,s,t,u,v,w}
O	20.7±0.7	10.2±0.7^{w,x}
P	21.8±0.4	9.7±0.4^{t,u,v,w,x}
Q	26.9±0.3	7.5±0.3^{n}
T	23.0±0.7	9.2±0.7^{p,q,r,s,t,u,v,w,x}
U	20.0±0.4	10.5±0.4^{x}
V	19.9±0.3	10.5±0.3^{x}
Z	22.9±0.3	9.2±0.3^{p,q,r,s,t,u,v,w,x}
A2	25.6±0.3	8.1±0.3^{m,n,o,p,q,r,s}
A3	22.6±0.3	9.3±0.3^{q,r,s,t,u,v,w,x}
A4	22.1±0.9	9.6±0.9^{s,t,u,v,w,x}
A5	22.0±0.3	9.6±0.3^{s,t,u,v,w,x}

Table S5. Continued

Bread code	Cycle threshold (C_T)	Gene copy number (Log gene copy/g)
	Sourdough breads	
A6	22.7±0.5	9.3±0.5^{q,r,s,t,u,v,w,x}
A7	21.4±0.2	9.9±0.2^{u,v,w,x}
A8	25.6±0.3	8.1±0.3^{m,n,o,p,q,r,s}
A9	24.9±0.6	8.4±0.6^{m,n,o,p,q,r,s,t,u}
A11	26.4±0.3	7.7±0.3^{m,n,o,p}
A16	23.4±0.3	9.0±0.3^{o,p,q,r,s,t,u,v,w,x}
A17	25.6±0.1	8.1±0.1^{m,n,o,p,q,r,s}
A18	29.9±0.8	6.2±0.8^{f,g,h,i,j}
A19	24.9±0.7	8.4±0.7^{m,n,o,p,q,r,s,t,u}
A21	25.4±0.8	8.2±0.8^{m,n,o,p,q,r,s,t}
A24	31.6±0.5	5.5±0.5^{e,f,g,h}
A26	25.8±0.2	8.0±0.2^{m,n,o,p,q,r}
A29	23.0±0.4	9.2±0.4^{p,q,r,s,t,u,v,w,x}
A31	23.0±0.1	9.2±0.1^{p,q,r,s,t,u,v,w,x}
A32	25.2±0.7	8.2±0.7^{m,n,o,p,q,r,s,t}
A37	28.8±0.6	6.7±0.6^{h,i,j,k}
A40	21.7±0.4	9.7±0.4^{t,u,v,w,x}
A41	23.2±0.9	9.1±0.9^{p,q,r,s,t,u,v,w,x}
A42	22.0±0.1	9.6±0.1^{s,t,u,v,w,x}
A43	30.0±0.5	6.2±0.5^{f,g,h,i,j}
A44	21.1±0.4	10.0±0.4^{v,w,x}

Table S5. Continued

Bread code	Cycle threshold (C_T)	Gene copy number (Log gene copy/g)
	Sourdough breads	
A47	24.9±0.4	$8.4\pm0.4^{m,n,o,p,q,r,s,t,u}$
A51	25.9±0.6	$7.9\pm0.6^{m,n,o,p,q}$
A52	25.5±0.1	$8.1\pm0.1^{m,n,o,p,q,r,s}$
2	24.6±0.3	$8.5\pm0.3^{n,o,p,q,r,s,t,u,v}$
6	26.2±0.9	$7.8\pm0.9^{m,n,o,p,q}$
8	25.2±0.2	$8.2\pm0.2^{m,n,o,p,q,r,s,t}$
11	28.0±0.3	$7.0\pm0.3^{i,j,k,l,m}$
12	25.0±0.3	$8.3\pm0.3^{m,n,o,p,q,r,s,t}$
13	24.4±0.2	$8.6\pm0.2^{n,o,p,q,r,s,t,u,v}$
14	22.6±0.2	$9.3\pm0.2^{q,r,s,t,u,v,w,x}$
15	24.6±0.3	$8.5\pm0.3^{n,o,p,q,r,s,t,u,v}$
26	23.4±0.6	$9.0\pm0.6^{o,p,q,r,s,t,u,v,w,x}$
27	22.1±0.4	$9.6\pm0.4^{s,t,u,v,w,x}$
28	26.6±0.2	$7.6\pm0.2^{m,n,o}$
29	23.1±0.6	$9.1\pm0.6^{p,q,r,s,t,u,v,w,x}$
32	21.2±0.3	$9.9\pm0.3^{u,v,w,x}$
	Baker’s yeast breads	
R	31.9±0.3	$5.3\pm0.3^{d,e,f,g,h}$
1	32.2±0.4	$5.2\pm0.4^{d,e,f,g,h}$
4	30.9±0.4	$5.7\pm0.4^{e,f,g,h,i}$
5	32.1±0.8	$5.2\pm0.8^{d,e,f,g,h}$

Table S5. Continued

**Bread code**	**Cycle threshold (C_T)**	**Gene copy number (Log gene copy/g)**
	Baker's yeast breads	
9	38.6±0.5	2.4±0.5^{a}
10	36.4±2.8	3.3±2.8^{a,b}
16	32.8±1.7	4.9±1.7^{d,e,f,g}
18	32.8±0.6	4.9±0.6^{d,e,f,g}
19	31.9±1.8	5.3±1.8^{d,e,f,g,h}
20	31.1±1.2	5.6±1.2^{e,f,g,h,i}
22	31.7±0.9	5.4±0.9^{d,e,f,g,h}
23	31.5±0.9	5.5±0.9^{e,f,g,h}
30	31.6±0.8	5.4±0.8^{d,e,f,g,h}
31	34.8±1.3	4.0±1.3^{b,c,d}
A10	33.4±1.2	4.6±1.2^{c,d,e}
A12	29.5±0.3	6.3±0.3^{g,h,i,j,k}
A13	32.4±1.0	5.1±1.0^{d,e,f,g}
A14	31.1±0.6	5.6±0.6^{e,f,g,h,i}
A15	30.7±0.6	5.8±0.6^{e,f,g,h,i}
A20	31.6±0.6	5.4±0.6^{d,e,f,g,h}
A22	32.1±0.8	5.2±0.8^{d,e,f,g,h}
A23	33.3±2.0	4.7±2.0^{c,d,e,f}
A25	31.3±0.3	5.5±0.3^{e,f,g,h}
A27	30.9±0.9	5.7±0.9^{e,f,g,h,i}
A28	31.5±0.5	5.5±0.5^{e,f,g,h}

Table S5. Continued

**Bread code**	**Cycle threshold (C_T)**	**Gene copy number (Log gene copy/g)**
	Baker's yeast breads	
A30	30.4±0.7	5.9±0.7^{e,f,g,h,i}
A33	36.1±1.2	3.4±1.2^{a,b,c}
A34	31.0±1.0	5.7±1.0^{e,f,g,h,i}
A35	31.0±0.8	5.7±0.8^{e,f,g,h,i}
A48	30.6±0.7	5.9±0.7^{e,f,g,h,i}
A49	32.9±0.4	4.8±0.4^{d,e,f,g}
A50	33.4±0.3	4.6±0.3^{b,c,d,e}
A45	29.5±0.2	5.8±0.2^{e,f,g,h,i}
A46	29.7±0.1	5.7±0.1^{e,f,g,h,i}
A39	31.1±0.8	5.6±0.8^{e,f,g,h,i}
A38	31.5±1.1	5.5±1.1^{e,f,g,h}

Description of commercial breads collected from bakeries are reported in Supplementary Table S6. Values are means ± standard deviation of three batches analysed in triplicate (n = 9). Values within a column with different superscript letters are significantly different (P<0.05).

Table S6. Description of commercial breads collected from bakeries

Bread code	Description	Commercial source
	Sourdough breads	
A	Whole grain “Senatore cappelli” wheat flour bead	Artisanal; Italy
B	Wheat sourdough. “Spelt bread”	Artisanal; Italy
C	Wheat sourdough. “Bread with almonds”	Artisanal; Italy
D	Wheat sourdough. “Khorasan bread”	Artisanal; Italy
E	Wheat sourdough. Whole grain bread” with “Senatore cappelli” wheat flour	Artisanal; Italy
I	Wheat sourdough. “Greek bread”	Artisanal; Italy
L	Wheat sourdough. Bread made with “Abbondanza” wheat flour	Artisanal; Italy
M	Wheat sourdough. “Bread with curcuma”	Artisanal; Italy
N	Wheat sourdough. “Bread with fruits”	Artisanal; Italy
O	Wheat sourdough. “Biological rye bread”	Artisanal; Italy
P	Wheat sourdough. “Bread with “Senatore cappelli” wheat flour	Artisanal; Italy
Q	Wheat sourdough Focaccia	Artisanal; Italy
T	Wheat sourdough bread	Artisanal; Italy
U	Wheat sourdough bread	Industrial; Italy
V	Wheat sourdough bread with fermented bran	Industrial; Italy
Z	Wheat sourdough bread with fermented bran&germ	Industrial; Italy
A1	Wheat sourdough bread	Artisanal; Italy
A2	Wheat sourdough bread	Artisanal; Italy
A3	Wheat sourdough bread	Artisanal; Italy
A4	Wheat sourdough Brioche	Artisanal; Italy
A5	Wheat sourdough bread	Industrial; Italy
A6	Wheat sourdough bread with fermented bran	Industrial; Italy
A7	Wheat sourdough bread with fermented bran&germ	Industrial; Italy
A8	Wheat sourdough. Altamura like- bread	Artisanal; Italy
A9	Wheat sourdough bread	Industrial; Italy
A11	Wheat sourdough sliced bread	Industrial; Italy
A16	Wheat firm sourdough bread	Industrial; Italy
A17	Wheat sourdough bread	Artisanal; Italy
A18	Wheat sourdough bread	Artisanal; Italy
A19	Wheat liquid sourdough bread	Industrial; Italy
A21	Wheat sourdough bread	Artisanal; Italy
A24	Wheat sourdough bread	Artisanal; Italy
A26	Wheat sourdough bread	Artisanal; Italy
A29	Wheat sourdough. “Barley bread”	Artisanal; Italy
A31	Wheat sourdough. “Buckwheat bread”	Artisanal; Italy
A32	Wheat sourdough sliced bread	Industrial; Italy
A37	Wheat sourdough bread	Artisanal; Italy
A40	Wheat sourdough bread	Industrial; Italy

Table S6. Continued

Bread code	Description	Commercial source
	Sourdough breads	
A41	Wheat sourdough bread	Industrial; Italy
A42	Wheat sourdough bread	Industrial; Italy
A43	Wheat sourdough bread	Artisanal; Italy
A44	Wheat sourdough bread	Artisanal; Italy
A47	Wheat sourdough “Pane di Altamura”	Artisanal; Italy
A51	Wheat sourdough puccia	Industrial; Italy
A52	Wheat sourdough puccia	Industrial; Italy
2	Strong wheat sourdough (100%) bread	Artisanal; Ireland
6	Baguette	Industrial; Ireland
8	Wheat sourdough. 70% rye & 30% wheat bread	Artisanal; Ireland
11	Mild wheat sourdough bread	Artisanal; Ireland
12	Brown sourdough. whole grain bread 55%	Artisanal; Ireland
13	Wheat sourdough bread (65%)	Artisanal; Ireland
14	Strong rye sourdough bread (100%)	Artisanal; Ireland
15	Rye sourdough (40%) bread	Artisanal; Ireland
26	Sourdough. 60% Rye&40% wheat	Artisanal; Ireland
27	Wheat sourdough. “Six grains bread”	Artisanal; Ireland
28	Mild 50% spelt & 50% rye	Artisanal; Ireland
29	French loaf wheat sourdough	Artisanal; Ireland
32	Sanfrancisco sourdough bread	Industrial; Ireland
	Bakers’ yeast breads	
G	Barley bread	Industrial; Italy
R	Sliced wheat bread	Industrial; Italy
H	Rye bread	Industrial; Italy
A10	White bread	Artisanal; Italy
A12	White bread	Artisanal; Italy
A13	White bread	Artisanal; Italy
A14	White bread	Artisanal; Italy
A15	White bread	Artisanal; Italy
A20	White bread	Artisanal; Italy
A22	White bread	Industrial; Italy
A23	White bread	Industrial; Italy
1	Jack Cuthbert's wheat bread	Industrial; Ireland
4	Wheat bread	Industrial; Ireland
5	O'Keeffe's wheat bread: sliced	Industrial; Ireland
9	White bread	Artisanal; Ireland
10	White bread	Artisanal; Ireland
16	White sliced bread	Industrial; Ireland
18	Healy's High fibre bran loaf brown	Industrial; Ireland
19	Multi-grains & seeds brown	Artisanal; Ireland

Table S6. Continued

Bread code	Description	Commercial source
	Baker’s yeast samples	
20	Brown bread	Artisanal; Ireland
22	Twomeys bakery wheat bread	Industrial; Ireland
23	O'Keeffe's wheat bread	Industrial; Ireland
30	Wheat sliced bread	Industrial; Ireland
31	White sliced bread	Industrial; Ireland
A25	White bread	Industrial; Italy
A27	White bread	Industrial; Italy
A28	White bread	Artisanal; Italy
A30	White sliced bread	Industrial; Italy
A33	White sliced bread	Industrial; Italy
A34	White sliced bread	Industrial; Italy
A35	Kamut bread	Artisanal; Italy
A48	White bread	Artisanal; Italy
A49	White bread	Artisanal; Italy
A50	White bread	Artisanal; Italy
A45	White bread	Artisanal; Italy
A46	White bread	Artisanal; Italy
A39	White bread	Artisanal; Italy
A38	White bread	Artisanal; Italy

Table S7. 16S rRNA gene-targeting primers from literature used in this study

**Primers**	**Sequence 5’ – 3’**	**References**
F_Lacto 05	AGC AGT AGG GAA TCT TCC A	Furet et al., 2009
R_Lacto 04	CGC CAC TGG TGT TCY TCC ATA TA	
α682F	CIA GTG TAG AGG TGA AAT T	De Gregoris et al., 2011
908αR	CCC CGT CAA TTC CTT TGA GTT	
1080γF	TCG TCA GCT CGT GTY GTG A	
γ1202R	CGT AAG GGC CAT GAT G	
928F-Firm	TGA AAC TYA AAG GAA TTG ACG	
1040FirmR	ACC ATG CAC CAC CTG TC	
Lac1 fw	AGC AGT AGG GAA TCT TCC A	Walter et al., 2001
Lac2	ATT YCA CCG CTA CAC ATG	
Lab-0677 rw	CAC CGC TAC ACA TGG AG	Heilig et al., 2002

Figure S1. Agarose gel electrophoresis. Agarose gel electrophoresis of the amplified genomic DNA from lactic acid bacteria and flour contaminants employing the newly designed primers as detailed in the accompanying text. M, ladder (Sigma-Aldrich, St. Louis, MO, USA); Lanes 1 and 2, *Lactobacillus fermentum* ATCC 14931 and F1; Lane 3, *Weissella confusa* DSM 20196; Lanes 4 and 5, *Pediococcus pentosaceus* PP5 and PP10; Lane 6, *Lactobacillus curvatus* ATCC 25601; Lane 7, *Leuonostoc citreum* 22A; Lanes 8 and 9, *Weissella cibaria* 5S, 7S; Lane 10, *Lactobacillus pentosus* ATCC 8041; Lanes 11 and 12, *Lactobacillus amylovarus* L_amy and ATCC 33620; Lanes 13 - 19, *Lactobacillus plantarum* DC400, POM1, RED3, 1M, 5-5 3DM, and; WCFS1; Lane 20, *Lactococcus lactis* 10γ, Lane 21, *Lactobacillus delbrueckii* B15Z; Lanes 22 - 24, *Lactobacillus rossiae* DSM 18514, LB5 and 8-5; Lanes 25 - 30, *Lactobacillus sanfranciscensis* 13, 7A, A1, E21 ,E14 and 274; Lane 31, *Lactobacillus brevis* LB4; Lanes 32 and 33, *Enterococcus faecalis* ATCC 23655 and 40; Lanes 34 and 35, *Enterococcus faecium* DSM 20477 and 22; Lane 36, *Lactobacillus hilgardii* ATCC 8290; Lane 37, *Lactobacillus hammesii* ATCC 23074; Lane 38, *Lactobacillus acidifarinae* DSM 19394; Lane 39, *Lactobacillus amylolyticus*; Lane 40, *Lactobacillus frumenti* DSM 13145; Lane 41, *Lactobacillus namurensis* DSM 19117; Lane 42, *Lactobacillus nantensis* DSM 16982; Lane 43, *Lactobacillus pontis* DSM 8475; Lane 44, *Lactobacillus panis* DSM 6035; Lane 45, *Lactobacillus crispatus* DSM 20584; Lane 46, *Lactobacillus farciminis* DSM 20184; Lane 47, *Leuconostoc mesenteroides* subsp. *cremoris*; Lane 48, *Leuconostoc mesenteroides* subsp. *dextranicum* DSM 46216; Lane 49 and 50, *Leuconostoc mesenteroides* subsp. *mesenteroides* DSM 20343 and LM5; Lane 51, *Lactobacillus alimentarius*; Lane 52, *Lactobacillus casei* ATCC-393; Lane 53, *Lactobacillus paralimentarius* DSM 13238; Lane 54, *Lactobacillus acidophilus* ATCC-4356; Lanes 55 and 56, *Lactobacillus kunkeei* LKA and; LKB; Lane 57, *Pediococcus acidilactici* DSM 20238; Lane 58, *Lactobacillus helveticus* ATCC 15009; Lane 59, *Lactobacillus reuteri* ATCC-23272; Lane 60, *Serratia marcescens*; Lanes 61 - 63, *Staphylococcus capitis* Sc1and *Sthaphylococcus* sp. S1 and Sta 41; Lanes 64 and 65, *Bacillus megaterium* Bm1 and *Bacillus* sp. B2; Lane 66, *Atlantibacter hermannii* Eh1; Lanes 67 - 69, *Pantoea agglomerans* Pag 37 and Pa3,

and *Pantoea* sp. Pan 38; Lanes 70 – 73, *Enterobacter aurogenes* Ea1, Ea2 and Ea3 and *Enterobacter* sp. E1; Lanes 74 and 75, *Pseudomonas fluorescent* Pf1 and *Pseudomonas* sp. P1; Lanes 76 and 77, *Acinetobacter calcoaceticus* Ac1 and *Acinetobacter* sp. A1; Lane 78, *Commamonas* sp. C1; Lane 79, *Erwinia* sp. Er1; Lane 80, *Sphingomonas* sp. Sp1; Lane 81, *Delftia* sp. D1; Lane 82, *Rhizobium* sp. R1; B, negative control. The numbers on the left indicate the molecular size of the DNA bands in base pairs.

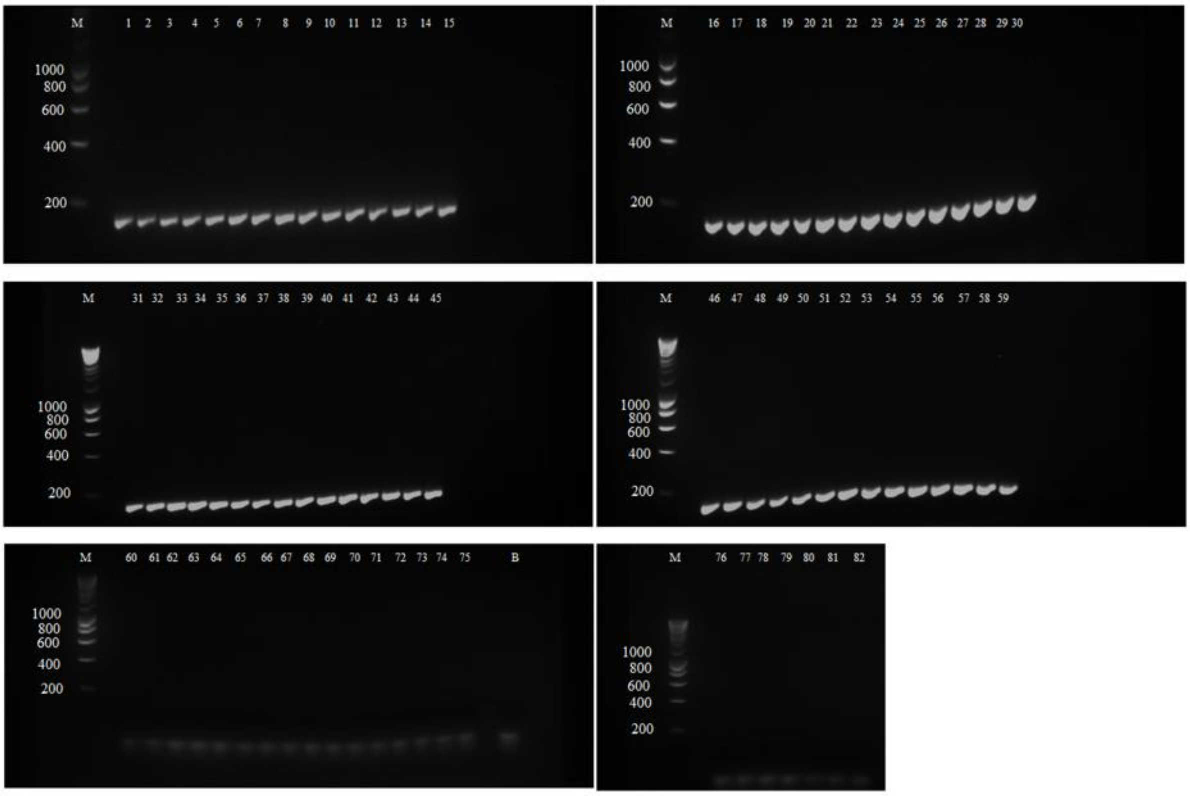

Figure S2. Calibration curve. Standard curve generated with the C_T values plotted against the logarithm of the 16S rRNA gene copies (Log copies/g) of *L. plantarum* WCFS1. DNA samples were extracted from breads inoculated with pure cultures of the strain (from 5.8 ± 0.2 to 9.8 ± 0.3 Log cfu/g). Data sets were subjected to one-way ANOVA; pair-comparison of treatment means was obtained by Tukey's procedure at P<0.05, using the statistical software Statistica 12.0 (StatSoft Inc., Tulsa, USA).

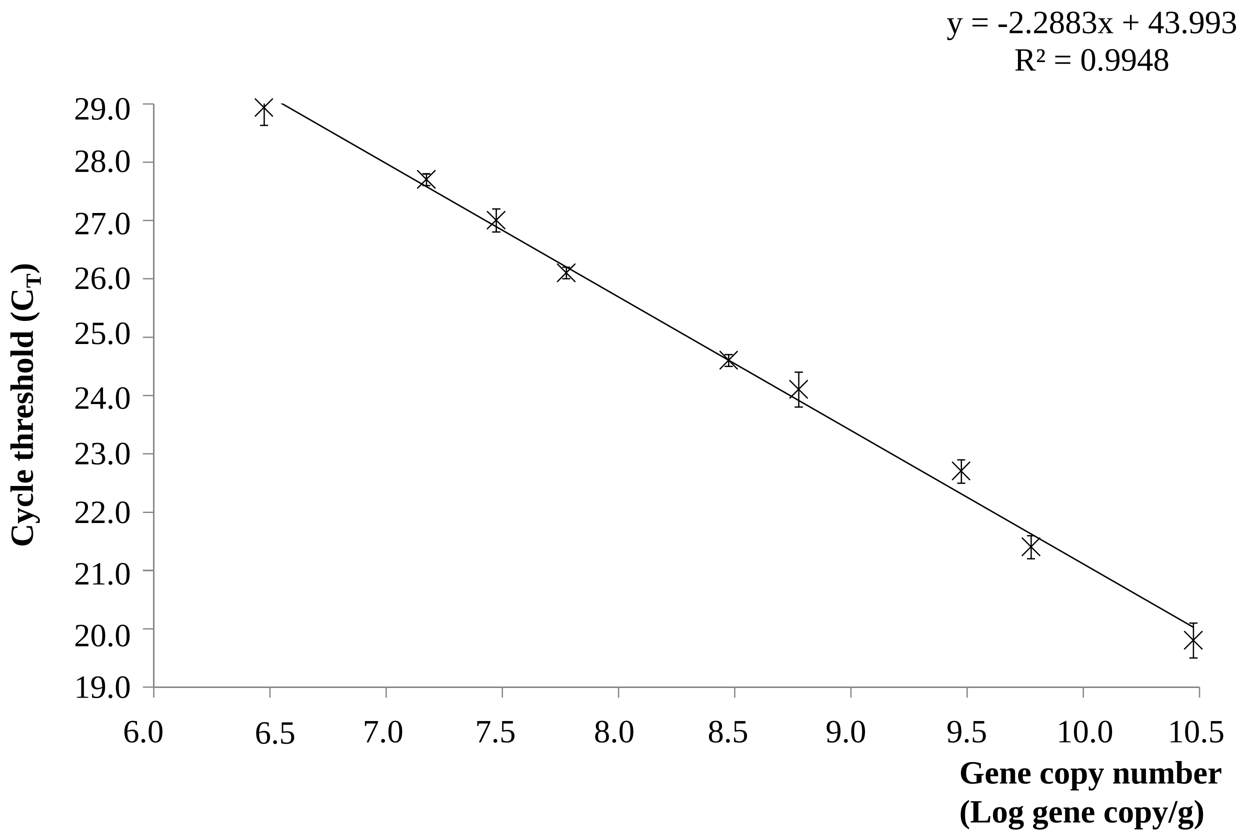

Supplement: Supplementary file 1 — Supplementary Information [file 41598_2017_549_MOESM1_ESM.pdf]
